# ﻿Passalidae (Coleoptera, Scarabaeoidea) from the Caribbean coast of Colombia: synopsis, key, and new species description

**DOI:** 10.3897/zookeys.1179.104037

**Published:** 2023-09-12

**Authors:** Larry Jiménez-Ferbans, Ana Maestre-Guerra, Evelin Villalba-Fuentes, Mayelis M. Barros-Barrios, Jeison Muñoz-Montero

**Affiliations:** 1 Facultad de Ciencias Básicas. Universidad del Magdalena, carrera 32 #22-08, Santa Marta, Zip code 470004, Colombia Universidad del Magdalena Santa Marta Colombia; 2 Facultad de Ciencias Exactas y Naturales. Universidad de Caldas, Calle 65 # 26- 10, Manizales, Zip code 170004, Colombia Universidad de Caldas Manizales Colombia

**Keywords:** Diversity, taxonomy, wood beetles

## Abstract

Bess beetles (Passalidae) are a subsocial family of Coleoptera with approximately 1000 known species of saproxylophagous diet and pantropical distribution, with few extratropical species. Because of their high levels of endemism (especially in mountains), feeding habits, and complex subsociability; Passalidae is considered an excellent biological subject for taxonomic, biogeographical, and evolutionary studies. Colombia is the richest country with more than 118 recorded species of Passalidae, most of the species being related to humid and mountain areas. Colombia’s Caribbean region constitutes the northern portion of the country, extending for more than 130,000 km^2^ and includes four of the eight biogeographical provinces of Colombia. Since the 2000s this region has been the subject of systematic surveys for Passalidae; as a result, 18 passalid species have been recorded to date. After new explorations and review of entomological collections, the knowledge of the passalid fauna for the region is updated, recording 28 species (8 new records, 2 new species) for which are provided species diagnoses, photographs, and a taxonomic key. The dry plain, characteristic of the lowlands, is dominated by widely distributed species such as *Passaluspunctiger* and *Passalusinterstitialis*, while the mountainous systems provide species of more restricted distributions, some of them endemic to the Colombian Caribbean.

## ﻿Introduction

Beetles of the family Passalidae are saproxylophagous diet, playing an important role in nutrient recycling (Castillo and Reyes-Castillo 2003). Adults establish multigenerational colonies inside rotting logs, on which they also feed. Although passalids are not eusocial, they exhibit intricate social relationships that include sound communication, in which up to 15 different signals have been detected that vary according to the context (e.g., stress, courtship). Approximately 1000 species are known worldwide, distributed mainly in tropical-humid zones (Schuster 1978; Boucher 2006), most of which exhibit small ranges (i.e., high degree of endemism). Recent studies suggest that the family Passalidae originated in Pangaea, more than 200 million years ago (Beza-Beza et al. 2021); however, the family has a markedly pantropical distribution, with few extratropical species.

The previously mentioned characteristics make Passalidae an excellent biological subject for ecological, biogeographical, taxonomic, and evolutionary studies. Consequently, in Colombia, Passalidae is one of the faunistically best-known families of Coleoptera, with an estimated 118 species distributed in the country, making it the country with the highest number of recorded Passalidae species in the world (Jiménez-Ferbans et al. 2018a). Maybe due to affinity with humid ecosystems and historical process, the biogeographic regions with the highest levels of species richness are the Chocó and Amazon provinces, with 41 and 24 species respectively (Amat-García and Reyes-Castillo 2007; Jiménez-Ferbans et al. 2018b). However, many regions have not been studied comprehensively and further exploration is likely to increase the known number of species, especially in mountainous areas, such as the Andes and Sierra Nevada de Santa Marta (SNSM), given the high levels of endemism typified by montane passalid species (Beza-Beza et al. 2021).

**Figure 1.**
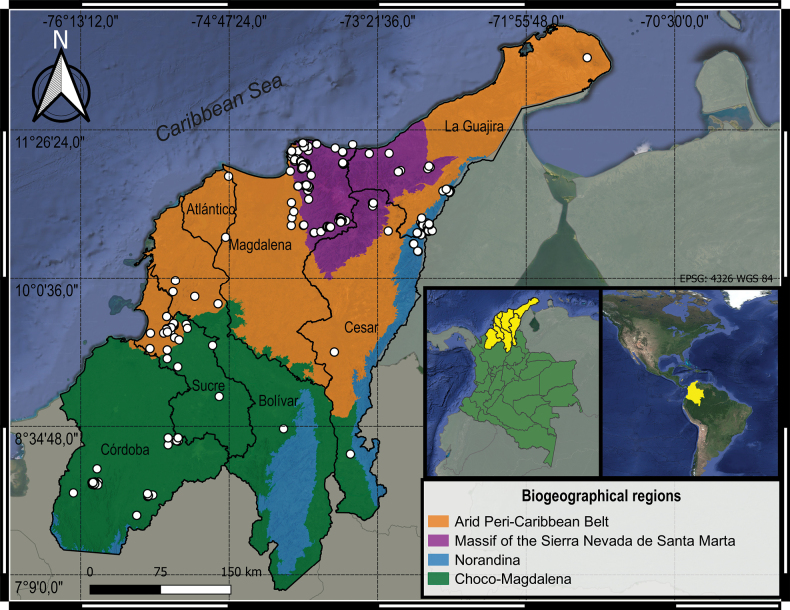
Map of the Colombian Caribbean region showing the biogeographical provinces according to Hernández-Camacho et al. (1992), and the localities with records from Passalidae.

The Colombian Caribbean is constituted, in geopolitical terms, by seven departments or states (La Guajira, Cesar, Magdalena, Atlántico, Bolívar, Sucre and Córdoba), covering more than 130,000 km^2^. It includes four of the eight main biogeographic provinces of Colombia (Fig. 1) and presents a high geomorphological heterogeneity that includes: savanna, tropical dry forest, and high Andean forest ecosystems, among others. Climatologically, there is a gradient of conditions in the Caribbean, starting in the northern zone (La Guajira and part of Magdalena) with arid, semi-arid, and semi-dry climates that decrease towards the south, until diverging into semi-humid, humid, and very humid climates in the departments of Bolivar, Cordoba, and Cesar (Carvajal-Cogollo and Rangel-Ch 2012).

The Caribbean region of Colombia is the most comprehensively know for passalids, based in amount of works published in the last two decades (e.g., Jiménez-Ferbans and Amat-García 2009; Jiménez-Ferbans et al. 2010; Jiménez-Ferbans et al. 2012, 2014, 2022; Taboada-Verona and Murillo-Ramos 2020). Jiménez-Ferbans and Amat-García (2009, 2010), and Jiménez-Ferbans et al. (2010) represented the first works that systematically studied the passalids of the region, registering 18 species and proposing a key and synopsis for their identification. Then, Jiménez-Ferbans et al. (2012, 2014, 2022) described new species for the Sierra Nevada de Santa Marta, increasing the number of species to 21. Additionally, recent explorations have allowed the discovery of species not registered for the region and increasing the number of sites with records of passalids. This work updates the knowledge of the family Passalidae in the Colombian Caribbean region, providing diagnoses, a taxonomic key, and the description of new species. A refined database of the revised material is also provided, with the aim of promoting the study of the family, facilitating the identification of species and the recognition of areas with exploration gaps.

## ﻿Materials and methods

We studied and digitized 5400 specimens deposited in Colección Entomológica Universidad del Magdalena (CBUMAG-ENT), Colombia (GBIF dataset: https://www.gbif.org/dataset/54F7AF87-E0A3-4816-AE9F-ED77C45F2455). We also included 380 records from Museo de Zoología de la Universidad de Sucre, Colombia (GBIF dataset https://www.gbif.org/dataset/6F1BED71-743E-407B-8862-E7E2E1CED896), and conducted a literature review. The total records, including those from literature, are included in a Darwin core formatted file (Suppl. material 1). GBIF data sets went through a quality process to guarantee its usability in research and decision-making processes, this was supported by the GBIF-BID “Data mobilization for key entomological groups across the Caribbean Region of Colombia”. For this, the datasets were verified in OpenRefine 3.7.2 (http://www.openrefine.org) and validated in GBIF Data Validator and the geographic validation service in QGIS provided by SiB Colombia (https://biodiversidad.co/formacion/laboratorios/QGIS). Taxonomic validation was performed according to the GBIF taxonomic tree.

The classification used is that of Beza-Beza et al. (2020) and Jiménez-Ferbans et al. (2023). While the terminologies of morphology are those proposed by Boucher (2006) for the head, except that we retain the term “frontal fossae” of Reyes-Castillo (1970), and the terminology of Reyes-Castillo (1970) for the rest of the body. For the taxonomic determinations, we employed the keys in Kuwert (1891), Marshall (2000), Boucher (2006), Gillogly (2005), Jiménez-Ferbans et al. (2018b); original descriptions and comparison with determined specimens deposited in collections. Total body length (based on the material examined) was measured from the apex of the mandible to the tip of the elytra with a digital caliper, when possible, we measured at least 10 specimens for each species.

For each species we examined, we give a diagnosis based on external morphological characters and photographs of relevant body areas. The photographs were taken using a Canon EOS Rebel SL3 camera. Then, they were stacked in layers by the software Helicon Focus v. 8.0.1 to generate a single image of combined focus. This image was edited for light and contrast correction in Adobe Photoshop, and the final combined figures were made using the same software, following the guide proposed by Bevilaqua (2020). Suppl. material 2 shows the label information for the photographed specimens.

## ﻿Results

The passalid fauna of the Colombian Caribbean Coast comprises 28 species of the genera *Passalus* (10 spp), *Rhodocanthopus* (2 spp), *Spasalus* (2 spp), *Paxilus* (1 spp), *Veturius* (4 spp), *Popilius* (3 spp), *Heliscus* (1 spp), *Odontotaenius* (1 spp), and *Verres* (2 spp) (Table 1). The biogeographical province of Chocó-Magdalena is the richest one, despite being the one with fewer locations with records, followed by the SNSM, which has the largest number of sampled localities (Table 2). As might be expected given the passalids preference for moist environments, the dry plain, characteristic of the lowlands of northern Colombia, has the least number of species, all of them being widely distributed.

**Table 1.** Species of Passalidae from the Colombian Caribbean Coast. The distribution indicates the department on the Caribbean Coast, followed by the general distribution of the species. Altitude data were taken from the material examined and ranges cited by other authors are given in parentheses.

Species	Distribution	Altitude (m)
Passalus (Passalus) coniferus	Magdalena. Argentina-northern Colombia	710–1880 (1550–1560)
Passalus (Passalus) interruptus	Córdoba, La Guajira, Magdalena, and Sucre. Argentina-northern Colombia	0–550 (375–2620)
Passalus (Passalus) interstitialis	Córdoba, La Guajira, Magdalena, and Sucre. Argentina-Mexico	0–480 (0–1500)
Passalus (Passalus) punctiger	Córdoba, Cesar, La Guajira, Magdalena, and Sucre. Argentina-Mexico	20–2260 (30–1500)
Passalus (Passalus) serankuai	Magdalena. Endemic to SNSM	1530–1950 (1530–1560)
Passalus (Passalus) chechai sp. nov.	Serranía del Perijá (La Guajira)	3019
Passalus (Passalus) florezi sp. nov.	Serranía del Perijá (La Guajira)	2460–2850
Passalus (Pertinax) gaboi	Magdalena. Endemic to SNSM	2040–2190 (1938–2190)
Passalus (Pertinax) paucuvillosus	Córdoba. Endemic to biogeographical province of Chocó	150–290 (0–550)
Passalus (Pertinax) punctatostriatus	Córdoba, Cesar, La Guajira, Magdalena, and Sucre. Northern Colombia-Mexico	120–2290 (0–2000)
Passalus (Pertinax) rugosus	La Guajira. Andes of Colombia	1900–2460 (270–2200)
Passalus (Pertinax) unimagdalenae	Cesar and Magdalena. Endemic to SNSM	710–2260 (1560–2309)
*Paxillusleachi*	Córdoba, Magdalena, and Sucre. Argentina-México	280–860 (250–1440)
*Rhodocanthopusmaillei*	La Guajira. Northern Andes	980–2280 (180–2400)
*Rhodocanthopusrufiventris*	Córdoba. Endemic to biogeographical province of Chocó	250–370 (50–160)
*Spasaluscrenatus*	Magdalena. South America and the Antilles.	400–1000
*Spasaluspaulinae*	Magdalena. South America-Puerto Rico	(1050)
*Heliscuseclipticus*	Córdoba. Northern South America-Mexico	170–260 (0–2438)
*Odontotaeniusstriatopunctatus*	Córdoba. Colombia-Mexico	341 (100–1200)
*Popiliuserotylus*	Córdoba and Magdalena. Northern Venezuela-Costa Rica	50–430 (250–750)
*Popiliusgibbosus*	Cesar and La Guajira. Andes	1520–2470 (1350–3000)
*Popiliusmarginatus*	Córdoba, Cesar, Magdalena, and Sucre. South America	870–1900 (250–1795)
*Verrescorticicola*	Córdoba. Northern South America-Mexico	240
*Verreshageni*	Córdoba. Northern South America-Mexico	200–500 (255–1500)
Veturius (Ouayana) cirratus	Córdoba. Ecuador-Costa Rica	130–540 (0–1000)
Veturius (Publius) impressus	Magdalena. Endemic to SNSM	1270–2050 (1560)
Veturius (Veturius) aspina	Córdoba and Sucre. Ecuador-Honduras	120–330 (0–1000)
Veturius (Veturius) standfussi	La Guajira. Andes of Bolivia-Venezuela	1280–1910 (800–2500)

**Table 2.** Richness and records of Passalidae by biogeographical province of the Caribbean Coast of Colombia. SNSM: Sierra Nevada de Santa Marta. Endemic species by province refer to those species endemic to a province and distributed in the Caribbean Coast.

Province	Species richness	Endemic species	Number of localities with records
Arid Peri-Caribbean belt	7		16
Massif of SNSM	14	4	43
Norandina	10	1	28
Chocó-Magdalena	15	2	13

### ﻿Synopsis of the species

#### Tribe Passalini

The species of this tribe are recognized by having the clypeus hidden under the frons, with anterior angles located under the external tubercles (Reyes-Castillo 1970) and frontoclypeus absent (Boucher 2006).

##### ﻿1. Passalus (Passalus) coniferus

Taxon classificationAnimaliaColeopteraPassalidae

Eschscholtz, 1829

0DC3CE5A-646E-541E-8D9E-650BE9185269

Fig. 2

###### Diagnosis.

39.6–46.5 mm total length. Body robust. Anterior border of the frons with two prominent secondary mediofrontal tubercles. Mediofrontal tubercles large, located on base of each laterofrontal tubercle. Central tubercle with apex free. Lateroposterior tubercles distinct. Eyes large. Antennal club tri-lamellate, with lamellae long. Lacinia with apex bidentate. Mediobasal area of mentum protruding and glabrous, sometimes with scarce setae on the posterior border. Marginal pronotal groove occupying 2/3 of the pronotum anterior border. Prosternellum rhomboidal, truncate. Mesosternum pubescent, with inconspicuous and elongated scars. Metasternum pubescent anterolaterally and in lateral groove; disc smooth, delimited posteriorly to middle by punctations. Humeri pubescent, epipleura pubescent in basal 2/3. Last abdominal sternite with complete marginal groove. Anterior ventral border of the profemur with well-developed groove. Meso- and metatibia with one or two small spines.

**Figure 2.**
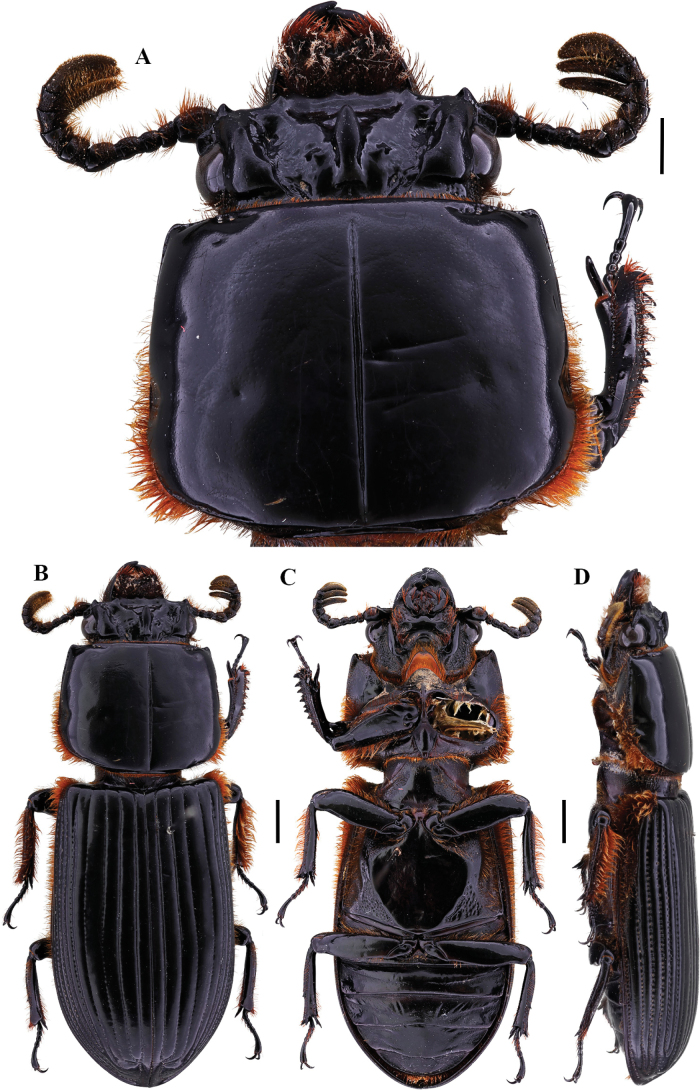
Passalus (Passalus) coniferus Eschscholtz, 1829 **A** head and pronotum in dorsal view **B** habitus dorsal **C** habitus ventral **D** habitus lateral. Scale bars: 2.0 mm (**A**); 3.0 mm (**B, C, D**).

###### Comments.

A South American species, with distribution in the Antilles (Reyes-Castillo 1973). It is a species of highly variable size, Reyes-Castillo and Amat-García (2003) notes that the specimens from the Sierra Nevada de Santa Marta are larger than those from the rest of the country, with the apex of the horn free and very long.

##### ﻿2. Passalus (Passalus) interruptus

Taxon classificationAnimaliaColeopteraPassalidae

(Linneus, 1758)

EE542A41-29DD-5D06-ADF4-3FC452E87F54

Fig. 3

###### Diagnosis.

41.2–52.7 mm total length. Body robust. Anterior border of the frons with two prominent secondary mediofrontal tubercles. Mediofrontal tubercles small, located on base of each laterofrontal tubercle. Central tubercle with apex slightly free. Lateroposterior tubercles distinct. Eyes large. Antennal club tri-lamellate, with lamellae long. Lacinia with apex bidentate. Mediobasal area of mentum protruding and glabrous. Marginal pronotal groove occupying 2/3 of the pronotum anterior border. Prosternellum rhomboidal, acute. Mesosternum with some sparse setae, with inconspicuous and elongated scars. Metasternum pubescent anterolaterally and in lateral groove; disc smooth and delimited by punctations posteriorly to middle. Humeri pubescent, epipleura pubescent in basal 2/3. Last abdominal sternite with incomplete marginal groove. Anterior ventral border of the profemur with conspicuous groove. Mesotibia with one or two small spines, metatibia unarmed.

**Figure 3.**
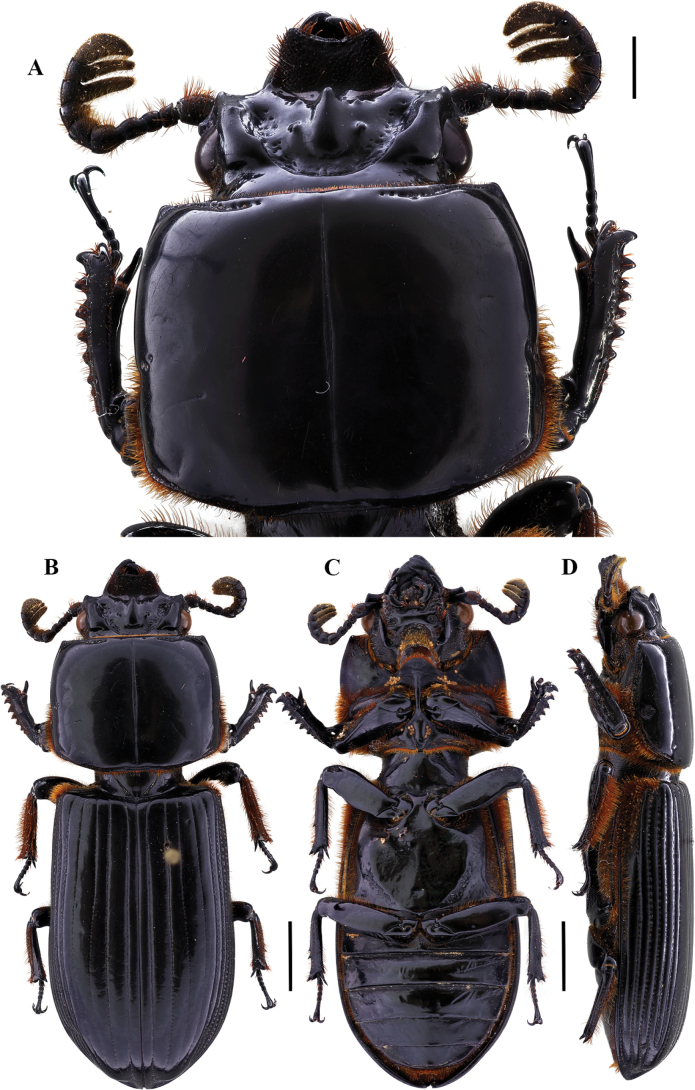
Passalus (Passalus) interruptus (Linneo, 1758) **A** head and pronotum in dorsal view **B** habitus dorsal **C** habitus ventral **D** habitus lateral. Scale bars: 3.0 mm (**A**); 5.0 mm (**B, C, D**).

###### Comments.

Distributed from Panama to Argentina and Trinidad and Tobago (Reyes-Castillo 1973). This species sometimes is confused with *P.punctiger*, but is distinguished by its larger size and incomplete marginal groove in last abdominal sternite (complete in *P.punctiger*).

##### ﻿3. Passalus (Passalus) interstitialis

Taxon classificationAnimaliaColeopteraPassalidae

Eschscholtz, 1829

E7663B3E-23B6-547C-A4B5-F9815E1BC446

Fig. 4

###### Diagnosis.

23.6–29.9 mm total length. Body flattened. Anterior border of the frons with two prominent secondary mediofrontal tubercles. Mediofrontal tubercles large, located on base of each laterofrontal tubercle. Central tubercle with apex not free. Lateroposterior tubercles distinct. Eyes large. Antennal club tetra-lamellate, fourth lamella reduced. Lacinia with apex bidentate. Mediobasal area of mentum glabrous and slightly protruding. Marginal pronotal groove occupying 2/3 of the pronotum anterior border. Prosternellum rhomboidal, truncate. Mesosternum glabrous, with distinct elongated scars. Metasternum pubescent anterolaterally and in lateral groove; disc smooth and delimited posteriorly to middle by punctations. Humeri pubescent, epipleura pubescent in basal third. Last abdominal sternite with complete marginal groove. Anterior ventral border of the profemur with distinct groove. Mesotibia lacking spine or with single small spine.

**Figure 4.**
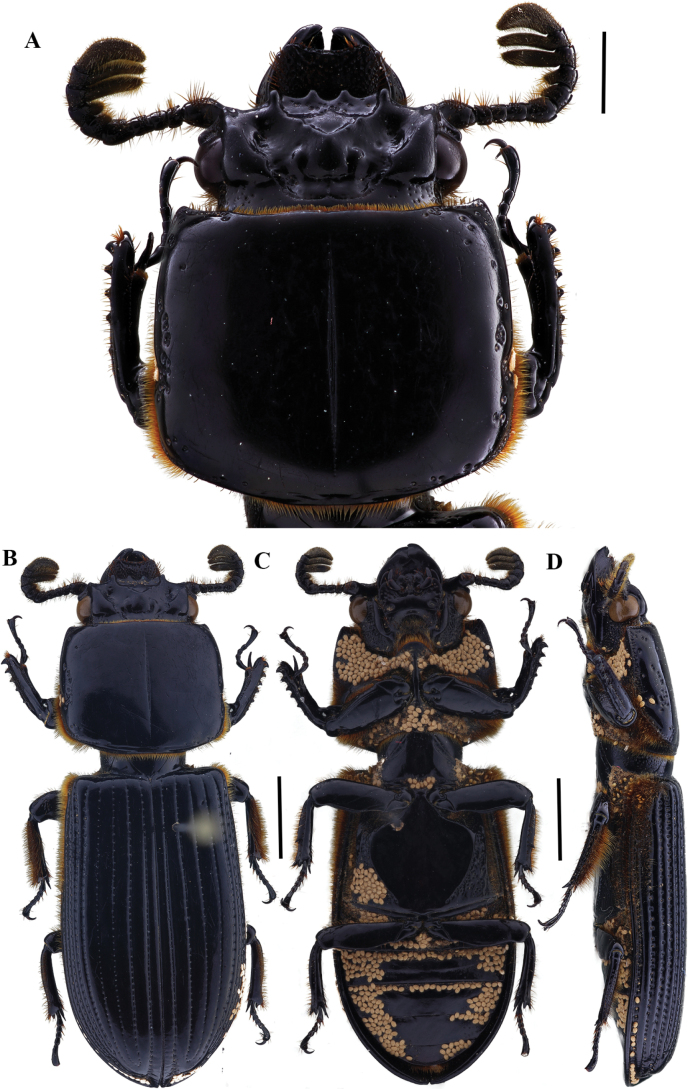
Passalus (Passalus) interstitialis Eschscholtz, 1829 **A** head and pronotum in dorsal view **B** habitus dorsal **C** habitus ventral **D** habitus lateral. Scale bars: 2.0 mm (**A**); 3.0 mm (**B, C, D**).

###### Comments.

Distributed from Mexico to Argentina (Reyes-Castillo 1973) and Cuba, Grenada, Jamaica, and Trinidad and Tobago (Jiménez-Ferbans et al. 2015). Sometimes confused with *P.punctiger*, *P.interstitialis* differs by its smaller size, flattened body, and apex of central tubercle not free.

##### ﻿4. Passalus (Passalus) punctiger

Taxon classificationAnimaliaColeopteraPassalidae

Lepeletier & Serville, 1825

0F19E65E-23DE-5756-9734-2B7B096FF29B

Fig. 5

###### Diagnosis.

28.4–41.7 mm total length. Body robust. Anterior border of the frons with two prominent secondary mediofrontal tubercles. Mediofrontal tubercles large, located on base of each laterofrontal tubercle. Central tubercle with apex slightly free. Lateroposterior tubercles conspicuous. Eyes large. Antennal club tri-lamellate, with lamellae long. Lacinia with apex bidentate. Mediobasal area of mentum protruding and glabrous. Marginal pronotal groove occupying 2/3 of the pronotum anterior border. Prosternellum rhomboidal, truncate. Mesosternum glabrous, with conspicuous and elongate scars. Metasternum pubescent anterolaterally and in lateral groove; disc smooth and delimited posteriorly to middle by punctations. Humeri pubescent, epipleura pubescent in basal 2/3. Last abdominal sternite with complete marginal groove. Anterior ventral border of the profemur with distinct groove. Meso- and metatibia with one or two small spines.

**Figure 5.**
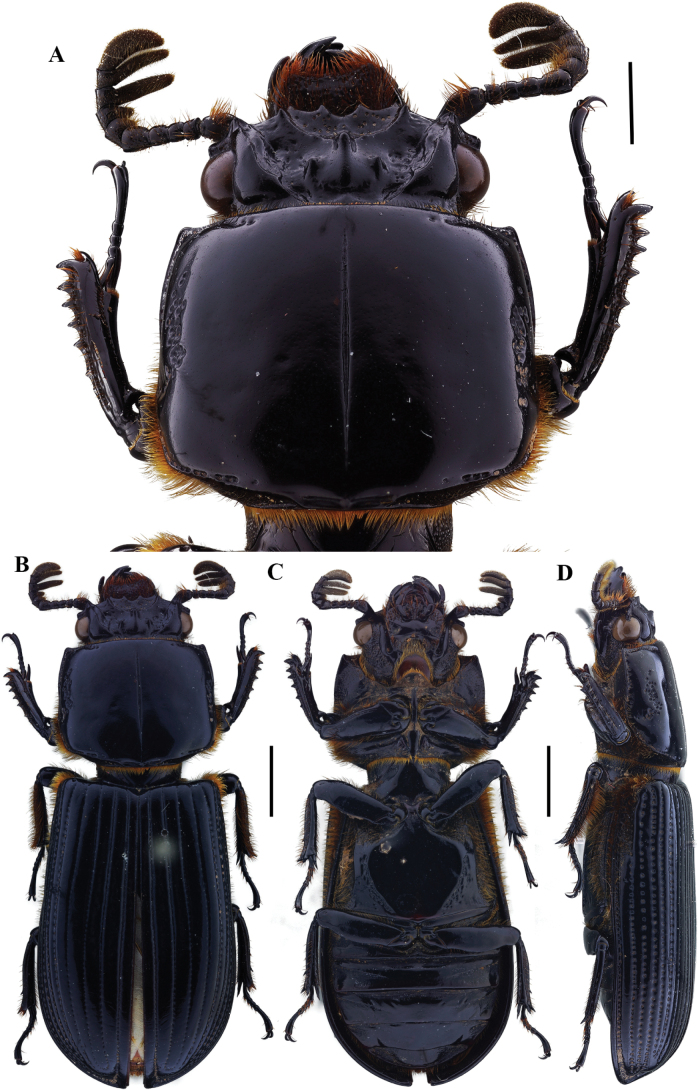
Passalus (Passalus) punctiger Lepeletier & Serville, 1825 **A** head and pronotum in dorsal view **B** habitus dorsal **C** habitus ventral **D** habitus lateral. Scale bars: 2.0 mm (**A**); 3.0 mm (**B, C, D**).

###### Comments.

Distributed from Mexico to Argentina. This the most common species for the lowlands of the Colombian Caribbean.

##### ﻿5. Passalus (Passalus) serankuai

Taxon classificationAnimaliaColeopteraPassalidae

Jiménez-Ferbans, Reyes-Castillo & Amat-García, 2014

02CB403D-DC47-533F-AC0A-390E9F4846A3

Fig. 6

###### Diagnosis.

28.7–34.2 mm total length. Body slightly flattened. Anterior border of frons with two prominent secondary mediofrontal tubercles, almost completely fused. Mediofrontal and laterofrontal fused, large. Central tubercle with apex very free, reaching or surpassing anterior frons border. Lateroposterior tubercles small, distinct. Eyes large. Antennal club tri-lamellate, with lamellae long. Lacinia with apex bidentate. Mediobasal area of mentum protruding and pubescent. Marginal pronotal groove occupying 2/3 of the pronotum anterior border. Prosternellum rhomboidal, truncate. Mesosternum glabrous, with distinct and elongate scars. Metasternum pubescent anterolaterally, lateral groove glabrous; disc smooth and delimited by punctations excluding the anterior part. Humeri pubescent, epipleura with some setae basally. Last abdominal sternite with complete marginal groove. Anterior ventral border of the profemur with conspicuous groove. Meso- and metatibia with small spines or unarmed.

**Figure 6.**
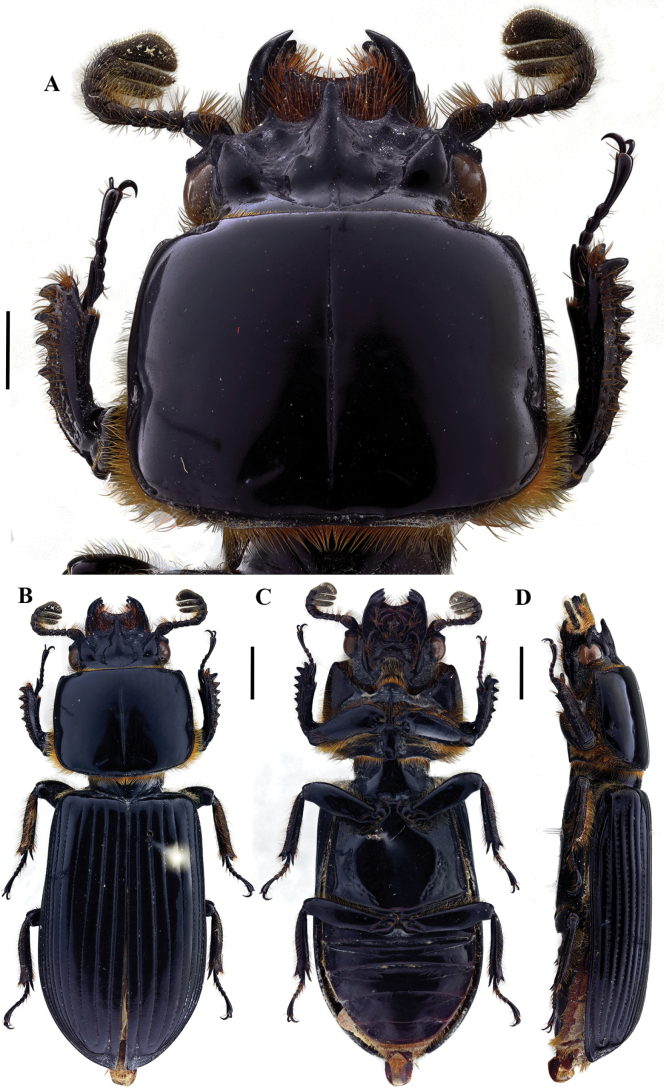
Passalus (Passalus) serankuai Jiménez-Ferbans Reyes-Castillo & Amat-García, 2014 **A** head and pronotum in dorsal view **B** habitus dorsal **C** habitus ventral **D** habitus lateral. Scale bars: 2.0 mm (**A**); 3.0 mm (**B, C, D**).

###### Comments.

Described from SNSM, this species seems to be endemic to this biogeographical province.

##### ﻿6. Passalus (Passalus) chechai

Taxon classificationAnimaliaColeopteraPassalidae

Jiménez-Ferbans sp. nov.

5CB4C5F6-23B1-5869-9551-E4D2D5167CDD

https://zoobank.org/DD10DD35-3D8D-4D4C-A162-EA5DCA40DC58

Figs 7 , 8

###### Type material.

***Holotype*** Colombia • ♂; La Guajira, Serranía del Perijá, Cerro Pintao; 10°25'37.9"N, 72°56'33.3"W; 3019 m alt.; 08 Aug 2015; L. Granados leg.; CBUMAG: ENT: 20605.

###### Diagnosis.

Hemibrachypterous. Frons wide, anterior frontal edge straight, without middle indentation or secondary mediofrontal tubercles. Central tubercle wide at the base, without a sulcus in the posterior part, apex free, almost reaching the anterior frons border. Medial basal mentum impunctate and glabrous. Prosternellum rhomboidal and opaque in the area between procoxae, without longitudinal groove. Anterolateral part of metasternum and lateral fossa pubescent, pubescence reaching the posterior region of the lateral fossa. Metasternal disc without punctures, delimited by numerous punctures posteriorly. Humeri pubescent and epipleura glabrous.

###### Description.

**Figure 7.**
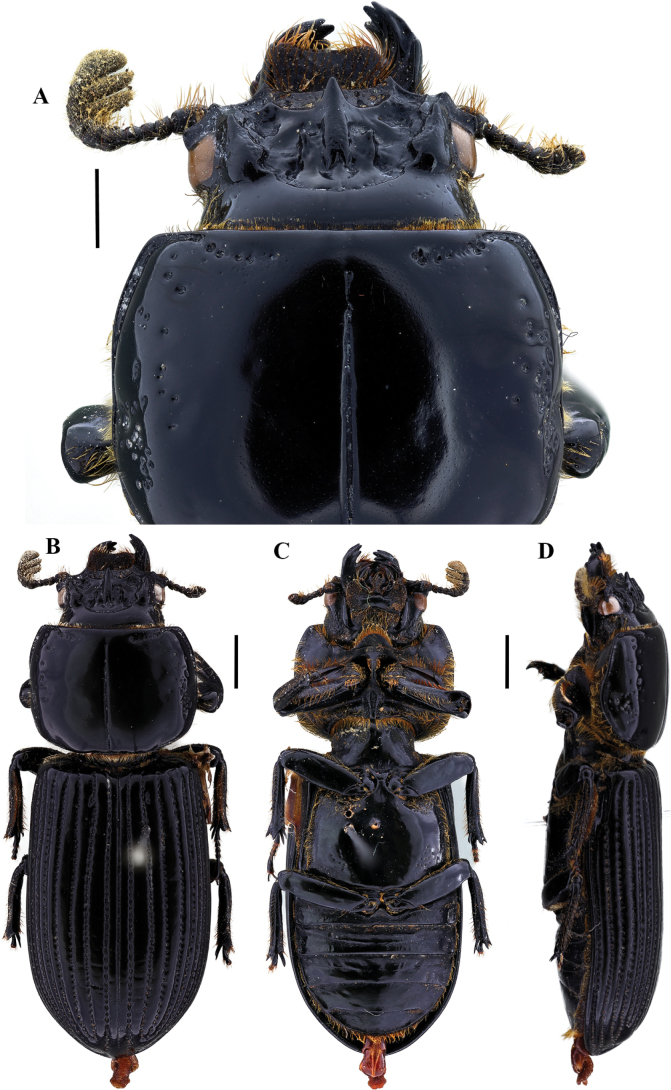
Passalus (Passalus) chechai sp. nov. **A** head and pronotum in dorsal view **B** habitus dorsal **C** habitus ventral **D** lateral view. Scale bars: 2.0 mm (**A**); 3.0 mm (**B, C, D**).

Habitus (Fig. 7B–D): total length 30.1 mm, hemibrachypterous, body convex shiny black.

**Figure 8.**
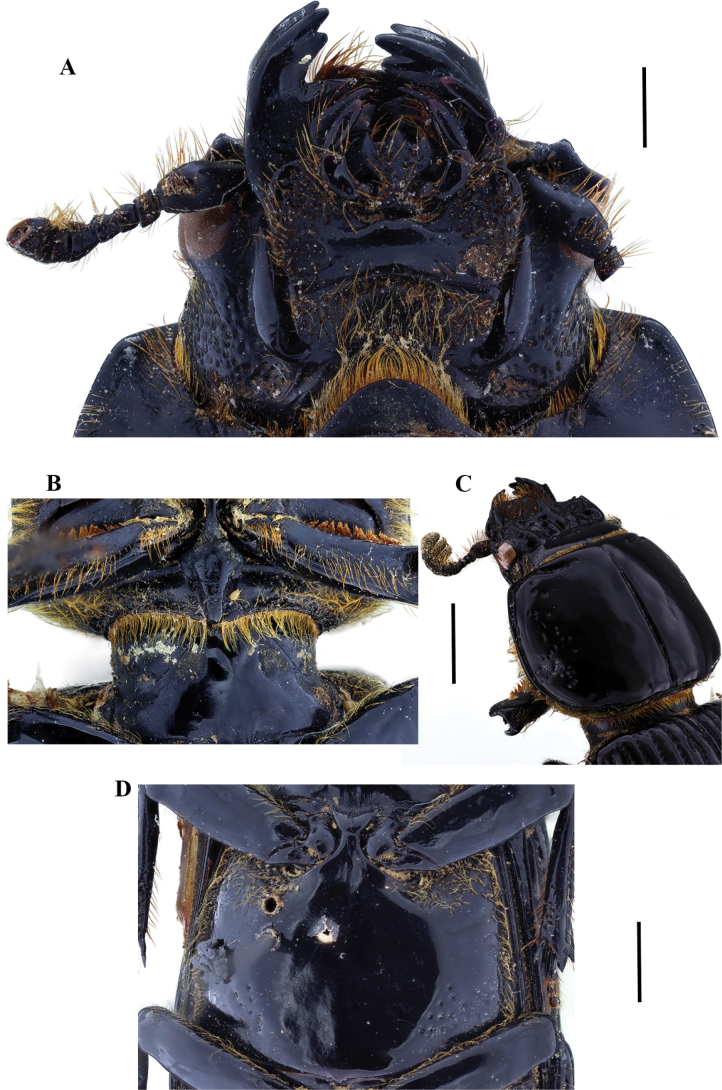
Passalus (Passalus) chechai sp. nov. **A** mentum **B** mesosternum **C** head and pronotum in dorso-lateral view **D** metasternum. Scale bars: 2.0 mm (**A, B, C, D**).

***Head*** (Figs 7A, D, 8A): labrum with anterior border slightly concave, evenly covered by setae. Clypeus hidden under the frons, anterior angles developed under the mediofrontal + laterofrontal tubercles and slightly smaller than these. Frons wide, anterior frontal edge straight, without middle indentation and secondary mediofrontal tubercles. Mediofrontal + laterofrontal tubercles projected forward, larger than internal tubercles. Internal tubercles small, joined to mediofrontal + laterofrontal tubercles by an inconspicuous ridge, placed at mid distance between the mediofrontal tubercles and the central tubercle base. Posterofrontal ridges “V” shaped. Area between the frontal ridges heavily punctuated without a median sulcus and cephalic mamelon (sensu Jiménez-Ferbans and Reyes-Castillo 2014). Mesofrontal structure of the “marginatus” type (Reyes-Castillo 1970), with central tubercle wide at the base, without a sulcus in the posterior part, apex free, almost reaching anterior frons border. Lateroposterior tubercles large, parallels to central tubercle. Lateropostfrontal areas glabrous, shiny, and impunctate. Eyes reduced, not extending past ocular canthi (dorsal view) and with canthus covering almost 1/2 of the eye in lateral view. Canthus glabrous. Postorbital pits shallow. Postfrontal groove semicircular and complete. Hypostomal process slightly separated from the mentum, glabrous and reaching the superior part of the middle zone of the mentum. Medial basal mentum protruding ventrally, impunctate and glabrous. Mentum with rounded lateral fossae, shallow and pubescent laterally. Antennal club tri-lamellate. Dorsal tooth straight on dorsal view and slightly sinuous on lateral view. Internal tooth of the left mandible bidentate, simple on the right mandible. Mandibular fossae short, not reaching the base of the mobile tooth. Maxilla with lacinia bidentate at the apex. Ligula tridentate, with middle tooth longer than the lateral teeth. Middle palpomere of the labial palp 1.3× wider and with almost the same length as the distal palpomere.

***Thorax*** (Figs 7, 8): Pronotum rounded, same width as elytra, with punctuations extending outside the lateral fossae and marginal groove. Marginal groove wide, occupying ¾ of the anterior margin of the pronotum. Longitudinal sulcus conspicuous. Lateral fossae distinct. Pre-epimeron shiny and heavily pubescent. Prosternellum rhomboidal and opaque in the area between procoxae. Mesosternum with erased mesosternal scars, indicated by an opaque area, impunctate and glabrous. Posterior corner of the mesepisternum and mesepimere glabrous and shiny. Anterolateral part of the metasternum and lateral fossa pubescent, pubescence reaching the posterior region of the lateral fossa. Metasternal disc without punctures, delimited by numerous punctures posteriorly. Posterior metasternal lateral fossa of the same width as epipleura.

***Elytra*** (Fig. 7B, D): Shiny, anterior border straight and pubescent. Humeri pubescent and epipleura glabrous. Striae with rounded punctures, equally distinctive on lateral and dorsal striae.

***Abdomen*** (Fig. 7C): Last sternite with marginal groove complete.

***Legs*** (Fig. 7C): profemur with ventral anterior marginal sulcus thin and complete, reaching the apical pubescence. Protibiae with dorsal sulcus complete. Meso- and metatibiae unarmed.

***Aedeagus*** (Fig. 7C, D): Basal piece (ventral view) fused with parameres and with deep V-shaped cleft. Median lobe globose, sclerotized on ventral surface, length 1× length of basal piece and parameres, measured at the median ventral line. Lateral projections of the parameres short and apex rounded in lateral view.

###### Etymology.

named after Mr. Cesar “Checha” Pérez, enthusiastic amateur collector of passalids in the Caribbean coast of Colombia.

##### ﻿7. Passalus (Passalus) florezi

Taxon classificationAnimaliaColeopteraPassalidae

Jiménez-Ferbans sp. nov.

F459D980-FF57-59BB-B3B3-AF34B8FDBD02

https://zoobank.org/5C17E16C-D5D5-437E-AC74-E5945AFC8BC2

Figs 9 , 10

###### Type material.

***Holotype*** Colombia • ♂; La Guajira, Villanueva, Serranía del Perijá, Cerro Pintao; 10°27'59.1"N, 72°52'4.3"W; 2467 m alt.; 19 Jan 2019; L. Jiménez-Ferbans and V. Flórez leg.; CBUMAG: ENT 33480. ***Paratypes***: Colombia • 3♀, 4♂; Same data as the holotype • 2♀; La Guajira, Villanueva, Serranía del Perijá, Cerro Pintao; 10°27'36.3"N, 72°52'11.0"W; 2845 m alt.; 19 Jan 2019; L. Jiménez-Ferbans and V. Flórez leg; CBUMAG:ENT 33150 • sex unknown; La Guajira, Villanueva, Serranía del Perijá, Cerro Pintao; 10°27'59.1"N, 72°52'4.3"W; 2467 m alt.; 19 Jan 2019; L. Jiménez-Ferbans and V. Flórez; CBUMAG:ENT 33147.

###### Diagnosis.

Brachypterous. Frons wide, anterior frontal edge straight, with middle indentation and secondary mediofrontal tubercles small (rudimentary). Central tubercle wide at the base, with a sulcus in the posterior part, apex free, reaching the anterior frons border. Medial basal mentum with punctures and setae on the posterior border. Prosternellum rhomboidal and opaque in the area between procoxae, with a deep longitudinal groove. Anterolateral part of metasternum with scarce pubescence, lateral fossa glabrous. Metasternal disc without punctures, delimited by numerous punctures medially and posteriorly. Humeri with scarce pubescence basally, epipleura glabrous.

###### Description.

**Figure 9.**
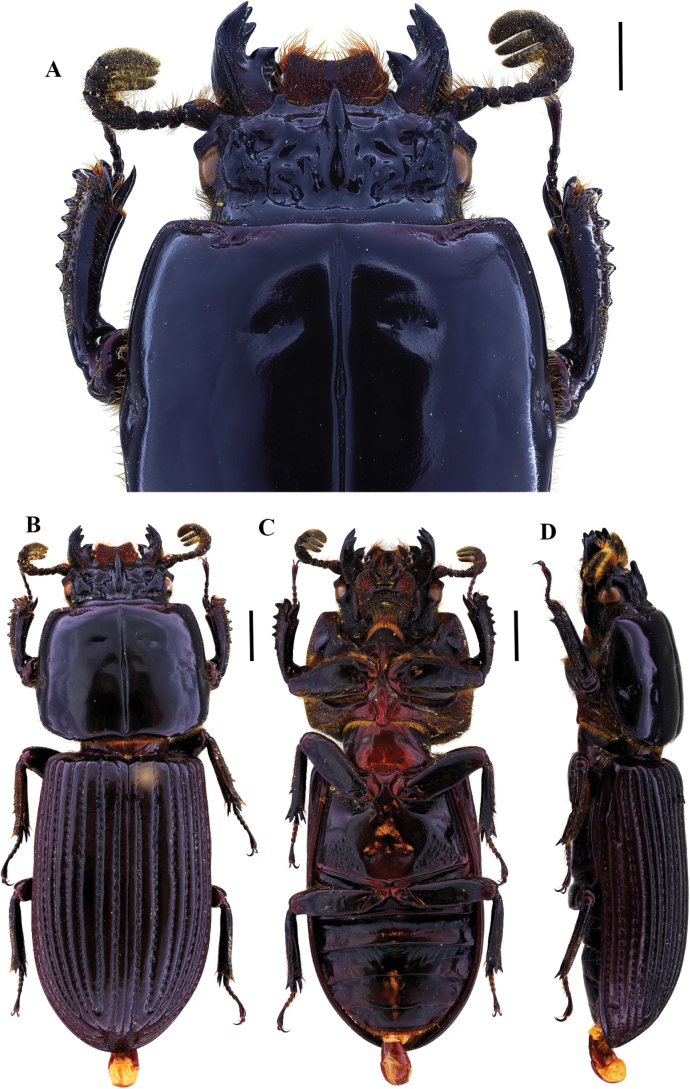
Passalus (Passalus) florezi sp. nov. **A** head and pronotum in dorsal view **B** habitus dorsal **C** habitus ventral **D** lateral view. Scale bars: 2.0 mm (**A**); 3.0 mm (**B, C, D**).

Habitus (Fig. 9B–D): total length 36.7–37.8 mm, hemibrachypterous, body convex shiny black.

**Figure 10.**
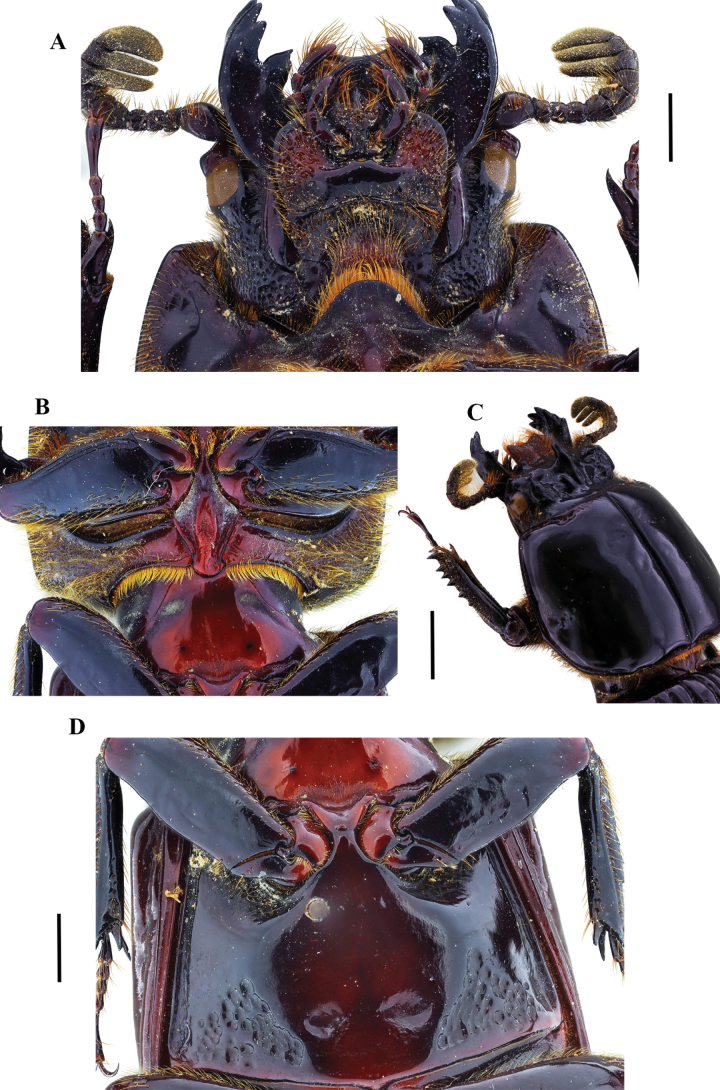
Passalus (Passalus) florezi sp. nov. **A** mentum **B** mesosternum **C** head and pronotum in dorso-lateral view **D** metasternum. Scale bars: 2.0 mm (**A, B, C, D**).

***Head*** (Figs 9A, D, 10A): labrum with anterior border concave, covered by setae less dense in the middle region. Clypeus hidden under the frons, anterior angles under the mediofrontal + laterofrontal tubercles and smaller than these. Frons wide, anterior frontal edge straight, with middle indentation and secondary mediofrontal tubercles rudimentary. Mediofrontal + laterofrontal tubercles projected forward, larger than internal tubercles. Internal tubercles small, rudimentary, joined to mediofrontal + laterofrontal tubercles by an inconspicuous ridge, placed at mid distance between the mediofrontal tubercles and the central tubercle base. Posterofrontal ridges V-shaped, inconspicuous. Area between the frontal ridges 3 punctures with a median sulcus and without cephalic mamelon (sensu Jiménez-Ferbans and Reyes-Castillo 2014). Mesofrontal structure of the “marginatus” type (Reyes-Castillo 1970), with central tubercle wide at the base, with a sulcus in the posterior part, apex free, reaching or surpassing anterior frons border. Lateroposterior tubercles large, parallel to central tubercle. Lateropostfrontal areas glabrous, shiny, and impunctate. Eyes reduced, not extending past ocular canthi (dorsal view) and with canthus covering almost 1/2 of the eye in lateral view. Canthus glabrous. Postorbital pits shallow. Postfrontal groove semicircular and complete. Hypostomal process slightly separated from mentum, glabrous and reaching the superior part of the middle zone of the mentum. Medial basal mentum protruding ventrally, with punctures and setae on posterior border. Mentum with rounded lateral fossae, shallow and pubescent laterally. Antennal club tri-lamellate. Dorsal tooth straight on dorsal view and slightly sinuous on lateral view. Internal tooth bidentate on left and right mandible. Mandibular fossa short, not reaching the base of the mobile tooth. Maxilla with lacinia bidentate at the apex. Ligula tridentate, with middle tooth longer than the lateral teeth. Middle palpomere of the labial palp 1.3× wider and with almost the same length as the distal palpomere.

***Thorax*** (Fig. 10B–D): Pronotum rounded, slightly wider than the elytra, with 3–10 punctures in or around lateral fossae. Marginal groove wide, occupying ¾ of the anterior margin of the pronotum. Longitudinal sulcus conspicuous. Lateral fossae distinct. Prepimerum shiny and pubescent. Prosternellum rhomboidal and opaque in the area between procoxae, with a deep longitudinal groove. Mesosternum with erased mesosternal scars, indicated by an opaque area, impunctate and glabrous. Posterior corner of the mesepisternum and mesepimere glabrous and opaque. Anterolateral part of metasternum with scarce pubescence, lateral fossa glabrous. Metasternal disc without punctures, delimited by numerous punctures medially and posteriorly. Posterior metasternal lateral fossa narrower than epipleura.

***Elytra*** (Fig. 9B, D): Shiny, anterior border straight and glabrous. Humeri with scarce pubescence basally, epipleura glabrous. Striae with rounded punctures, more distinctive on lateral striae.

***Abdomen*** (Fig. 9C): Last sternite with marginal groove incomplete.

***Legs*** (Fig. 9C): profemur with ventral anterior marginal sulcus thin and complete, reaching the apical pubescence. Protibiae with dorsal sulcus complete. Mesotibiae with a small spine on the outer margin. Metatibiae unarmed.

***Aedeagus*** (Fig. 9C, D): Basal piece (ventral view) fully fused with parameres and with deep V-shape cleft. Median lobe globose, little sclerotized on the ventral surface, length is 1.1× the length of the basal piece and parameres, measured at the median ventral line. Lateral projections of the parameres short and apex rounded on lateral view.

***Variation***: In some paratypes, the area between the frontal ridges has 0–4 punctures, and the metasternal disc with 0–10 punctures.

###### Etymology.

Named after Mr. Víctor Flórez Carrillo, for his dedication to entomological exploration in the Serranía del Perijá.

##### ﻿8. Passalus (Pertinax) gaboi

Taxon classificationAnimaliaColeopteraPassalidae

Jiménez-Ferbans & Reyes-Castillo, 2022

EC4C7F22-CD2C-50CF-84D3-8103BCFE37F2

Fig. 11

###### Diagnosis.

31.6–34.2 mm total length. Body robust. Anterior border of the frons with small middle indentation, without secondary mediofrontal tubercles. Mediofrontal and laterofrontal fused, midsize. Central tubercle with apex not free. Lateroposterior tubercles slightly distinct and rounded. Eyes reduced, with canthus covering 1/2 of the eye in lateral view. Antennal club tri-lamellate, with lamellae long. Lacinia with apex bidentate. Mediobasal area of mentum protruding and heavily punctate and pubescent. Marginal groove wide, occupying 3/4 of the anterior margin of the pronotum. Prosternellum rhomboidal, truncate. Mesosternum without mesosternal scars, indicated only by an opaque area, impunctate and glabrous. Metasternum pubescent anterolaterally and in lateral groove; disc smooth and delimited by numerous punctations medially and posteriorly. Humeri and epipleura glabrous. Last abdominal sternite with complete marginal groove. Anterior ventral border of the profemur with thin groove. Mesotibiae with small spines on the outer margin. Metatibiae unarmed.

**Figure 11.**
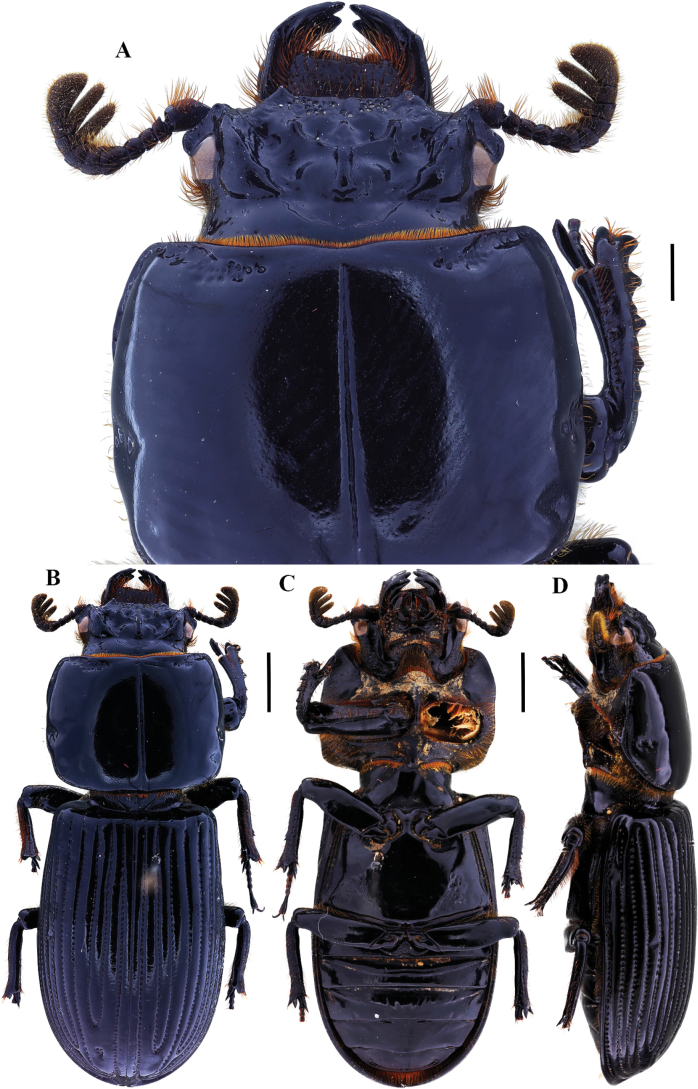
Passalus (Pertinax) gaboi Jiménez-Ferbans & Reyes-Castillo, 2022 **A** head and pronotum in dorsal view **B** habitus dorsal **C** habitus ventral **D** habitus lateral. Scale bars: 2.0 mm (**A**); 3.0 mm (**B, C, D**).

###### Comments.

*Passalusgaboi* had been the last species described from the Colombian Caribbean. It seems to be endemic to the Sierra Nevada de Santa Marta.

##### ﻿9. Passalus (Pertinax) paucuvillosus

Taxon classificationAnimaliaColeopteraPassalidae

Jiménez-Ferbans, Reyes-Castillo & Schuster, 2018

10A7DED0-BC84-5869-93DB-71E8A8647878

Fig. 12

###### Diagnosis.

22.9–25.5 mm total length. Body robust. Anterior border of the frons straight, with small and irregular tubercles, without secondary mediofrontal tubercles. Mediofrontal and laterofrontal fused, small, projected forward. Central tubercle small, with apex not free. Lateroposterior tubercles small, slightly distinct. Eyes large. Antennal club tri-lamellate, with lamellae short. Lacinia with apex bidentate. Mediobasal area of mentum protruding and glabrous. Marginal groove widened, occupying 2/3 of the anterior margin of the pronotum. Prosternellum rhomboidal, truncate. Mesosternum glabrous; mesosternal scars absent. Metasternum glabrous anterolaterally and in lateral groove; disc smooth and delimited by punctations posteriorly. Humeri and epipleura glabrous. Anterior ventral border of the profemur with thin groove. Meso- and metatibiae with small spines or unarmed.

**Figure 12.**
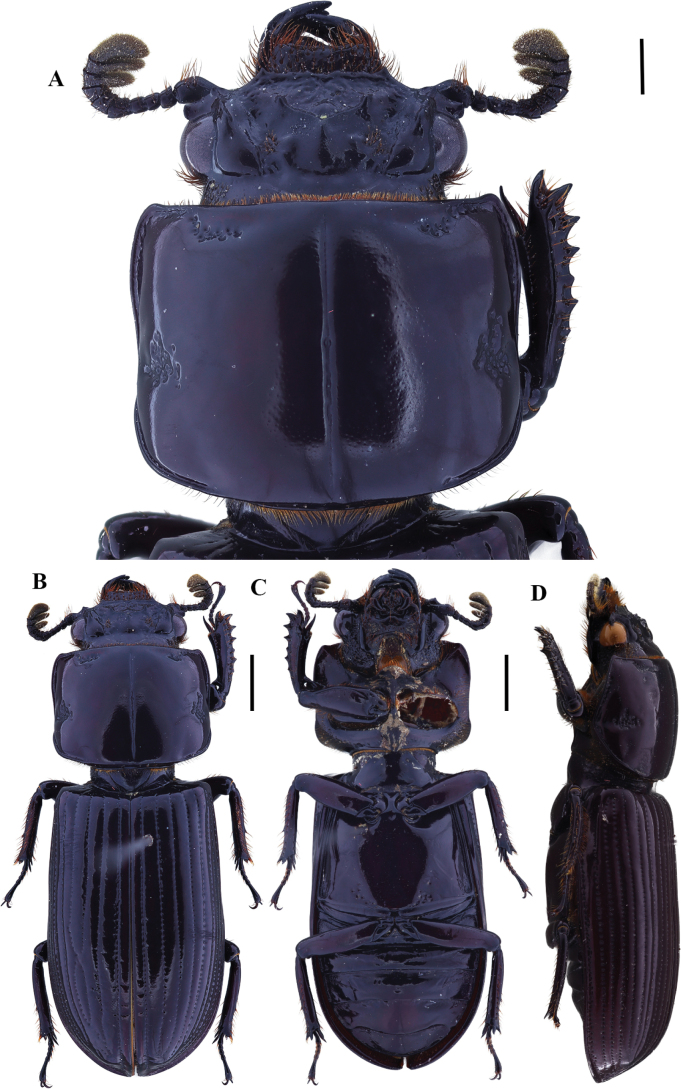
Passalus (Pertinax) paucuvillosus Jiménez-Ferbans, Reyes-Castillo & Schuster, 2018 **A** head and pronotum in dorsal view **B** habitus dorsal **C** habitus ventral **D** habitus lateral. Scale bars: 1.0 mm (**A**); 2.0 mm (**B, C, D**).

###### Comments.

Described from the Chocó department of Colombia (Jiménez-Ferbans et al. 2018b), this species seems to be endemic to the Chocó-Magdalena province.

##### ﻿10. Passalus (Pertinax) punctatostriatus

Taxon classificationAnimaliaColeopteraPassalidae

Percheron, 1835

04FB106E-30AA-57B2-9110-7941C7860C2A

Fig. 13

###### Diagnosis.

21.8–26.0 mm total length. Body subcylindrical. Anterior border of the frons with strong middle indentation, without secondary mediofrontal tubercles. Mediofrontal and laterofrontal fused, large. Central tubercle with apex not free. Lateroposterior tubercles small, slightly distinct. Eyes large. Antennal club tri-lamellate, with lamellae long. Lacinia with apex bidentate. Mediobasal area of mentum protruding and glabrous. Marginal groove widened, occupying 2/3 of the anterior margin of the pronotum. Prosternellum rhomboidal, acute. Mesosternum glabrous; mesosternal scars inconspicuous and elongated. Metasternum glabrous anterolaterally and in lateral groove; disc smooth and delimited by punctations posteriorly to laterally. Humeri glabrous and epipleura with some scarce setae basally or glabrous. Anterior ventral border of the profemur with groove. Meso- and metatibiae with small spines or unarmed.

**Figure 13.**
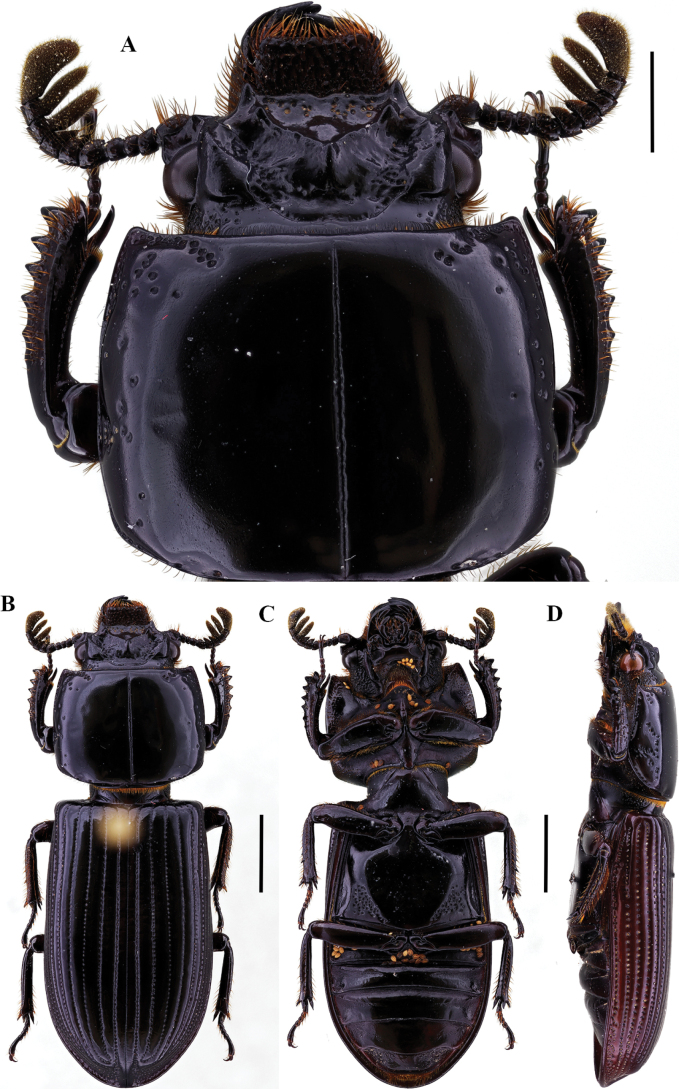
Passalus (Pertinax) punctatostriatus Percheron, 1835 **A** head and pronotum in dorsal view **B** habitus dorsal **C** habitus ventral **D** habitus lateral. Scale bars: 2.0 mm (**A**); 3.0 mm (**B, C, D**).

###### Comments.

Distributed from Mexico to central and northern Colombia. Amat-García et al. (2004) cited this species from the Amazon region, but without citing specimens. We doubt its presence in southern Colombia. *P.punctatostriatus* has been collected in shady coffee plantations in Serranía del Perijá and in Sierra Nevada de Santa Marta (Jiménez-Ferbans and Amat-García 2009).

##### ﻿11. Passalus (Pertinax) rugosus

Taxon classificationAnimaliaColeopteraPassalidae

Gravely, 1918

110085C5-84D4-5F61-9B32-7B1DA0591F04

Fig. 14

###### Diagnosis.

20.1–23.2 mm total length. Body subcylindrical. Anterior border of the frons with strong middle indentation, suggesting secondary mediofrontal tubercles. Mediofrontal and laterofrontal fused, large. Central tubercle with apex not free. Lateroposterior tubercles large, conspicuous, and conical. Eyes large. Antennal club tri-lamellate, with lamellae long. Lacinia with apex bidentate. Mediobasal area of mentum protruding and glabrous. Marginal groove narrow, occupying 2/3 of the anterior margin of the pronotum. Prosternellum rhomboidal, acute. Mesosternum glabrous; mesosternal scars distinct and elongated. Metasternum glabrous anterolaterally and in lateral groove; disc smooth and delimited by punctations posteriorly to laterally. Humeri pubescent and epipleura pubescent in basal third. Anterior ventral border of the profemur with conspicuous groove. Meso- and metatibiae with small spines or unarmed.

**Figure 14.**
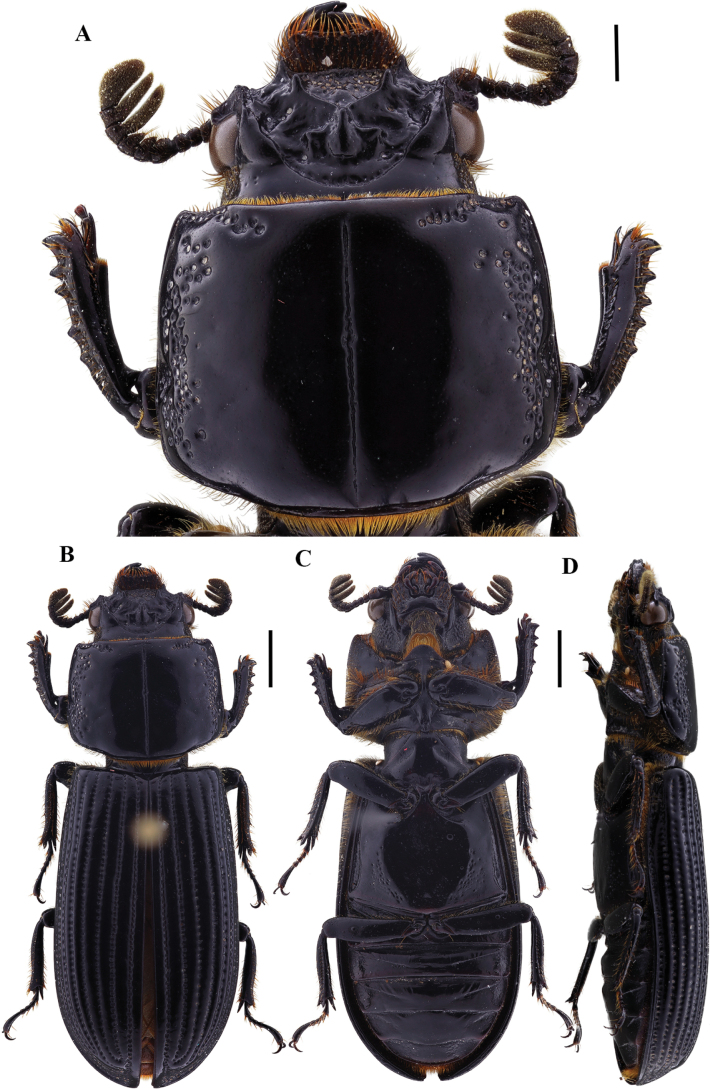
Passalus (Pertinax) rugosus Gravely, 1918 **A** head and pronotum in dorsal view **B** habitus dorsal **C** habitus ventral **D** habitus lateral. Scale bars: 1.0 mm (**A**); 2.0 mm (**B, C, D**).

###### Comments.

Jiménez-Ferbans et al. (2018b) considered this species as endemic to the western Andes of Colombia. However, the present records extend its distribution to the eastern Andes.

##### ﻿12. Passalus (Pertinax) unimagdalenae

Taxon classificationAnimaliaColeopteraPassalidae

Jiménez-Ferbans, Reyes-Castillo & Amat-García, 2012

0C660F3B-B8A0-50BC-8733-7ED7E28A57D4

Fig. 15

###### Diagnosis.

19.5–22.4 mm total length. Body subcylindrical. Anterior border of the frons with strong middle indentation, without secondary mediofrontal tubercles. Mediofrontal and laterofrontal fused, large. Central tubercle with apex not free. Lateroposterior tubercles small, slightly distinct. Eyes large. Antennal club tri-lamellate, with lamellae long. Lacinia with apex bidentate. Mediobasal area of mentum protruding and glabrous. Marginal groove widened, occupying 2/3 of the anterior margin of the pronotum. Prosternellum rhomboidal, truncate. Mesosternum glabrous, except for a few short setae on the anterior margin; mesosternal scars inconspicuous and elongated. Metasternum glabrous anterolaterally and in lateral groove; disc smooth and delimited by punctations posteriorly to laterally. Humeri with long setae at its base, epipleura glabrous. Anterior ventral border of the profemur with conspicuous groove. Meso- and metatibiae with small spines or unarmed.

**Figure 15.**
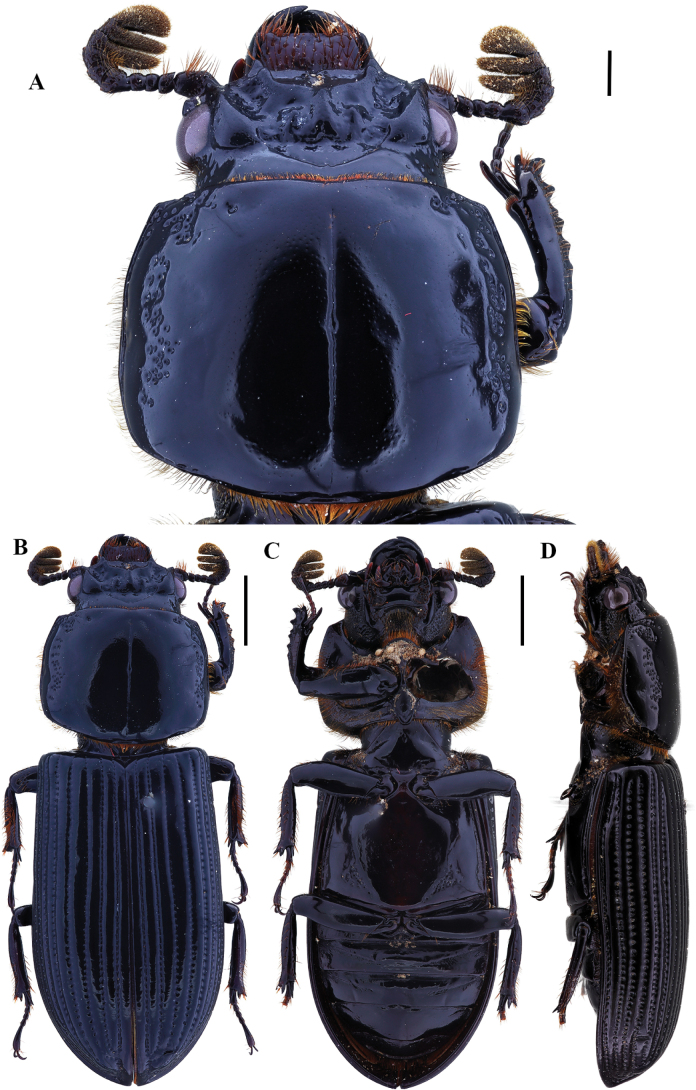
Passalus (Pertinax) unimagdalenae Jiménez-Ferbans, Reyes-Castillo & Amat-García, 2012 **A** head and pronotum in dorsal view **B** habitus dorsal **C** habitus ventral **D** habitus lateral. Scale bars: 1.0 mm (**A**); 2.0 mm (**B, C, D**).

###### Comments.

This is the most common species above 1500 m a.s.l. in Sierra Nevada de Santa Marta. *P.unimagdalenae* is similar to *P.punctatostriatus*, from which it differs by having internal tubercles large and humeri with long setae at the very base.

##### ﻿13. Paxillus leachi

Taxon classificationAnimaliaColeopteraPassalidae

MacLeay, 1819

F07B5143-E7DC-56B9-8CBA-CD5FCAB5D371

Fig. 16

###### Diagnosis.

17.5–18.8 mm total length. Anterior border of the frons straight, sometimes with a small central notch, without secondary mediofrontal tubercles. Mediofrontal tubercles large. Internal tubercules smaller than mediofrontal tubercles, located between mediofrontal tubercles and central tubercle. Central tubercle with apex not free. Lateroposterior tubercles distinct. Anterior cephalic angles reach the border of the frons. Frontal fossae glabrous. Eyes large. Antennal club with five lamellae, with the basal lamella no more than half as long as the apical four. Lacinia with apex unidentate. Mediobasal area of mentum flat and glabrous. Prosternellum pentagonal. Mesosternum glabrous, with conspicuous oval scars. Metasternum pubescent anterolaterally and glabrous in lateral groove; disc smooth, completely delimited by punctures. Humeri pubescent and epipleura pubescent in basal 1/4. Anterior ventral border of the profemur without groove or inconspicuous.

**Figure 16.**
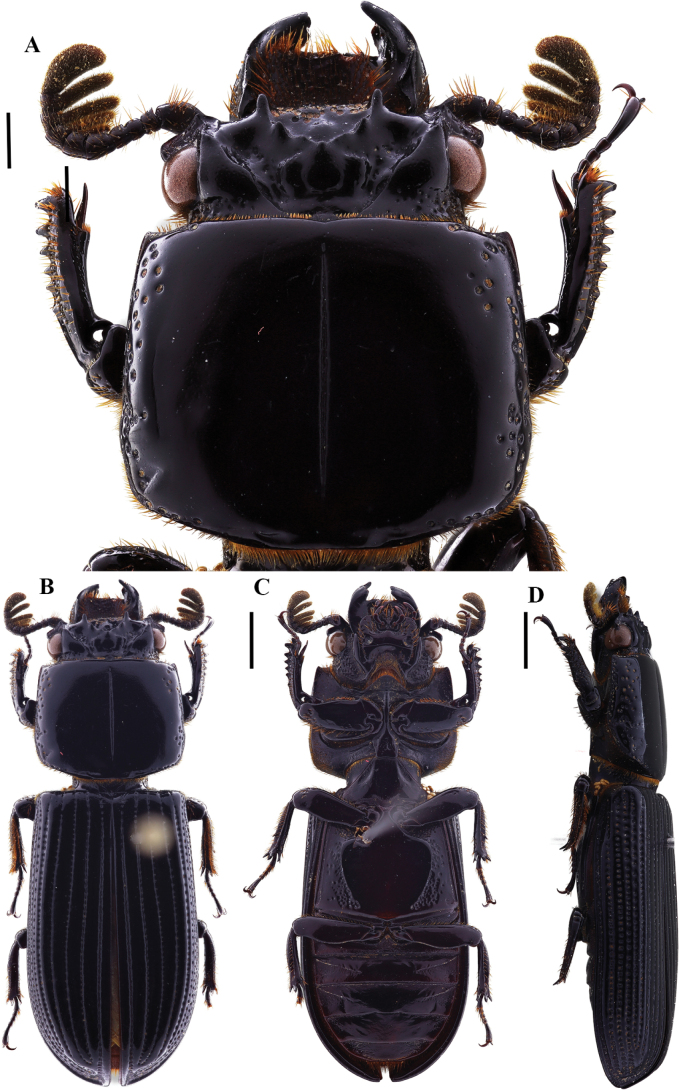
*Paxillusleachi* MacLeay, 1819 **A** head and pronotum in dorsal view **B** habitus dorsal **C** habitus ventral **D** habitus lateral. Scale bars: 1.0 mm (**A**); 2.0 mm (**B, C, D**).

###### Comments.

This species is distributed throughout the American continent, from Mexico to Argentina.

##### ﻿14. Rhodocanthopus maillei

Taxon classificationAnimaliaColeopteraPassalidae

(Percheron, 1841)

9B16DF7E-A0F9-527C-9F80-81D63AB44C4A

Fig. 17

###### Diagnosis.

16.3–19.8 mm total length. Body subcylindrical. Anterior border of the frons with strong middle indentation, without secondary mediofrontal tubercles. Mediofrontal and laterofrontal fused, large. Central tubercle with apex not free. Lateroposterior tubercles conspicuous and conical. Eyes reduced. Antennal club tri-lamellate, lamellae short. Lacinia with apex bidentate. Mediobasal area of mentum protruding and glabrous. Marginal groove widened, occupying 2/3 of the anterior margin of the pronotum. Prosternellum rhomboidal, acute. Mesosternum glabrous; mesosternal scars oval and distinct. Metasternum glabrous anterolaterally and in lateral groove; disc smooth and delimited by punctations posteriorly to laterally. Humeri and epipleura glabrous. Anterior ventral border of the profemur with conspicuous groove. Meso- and metatibiae with strong spines.

**Figure 17.**
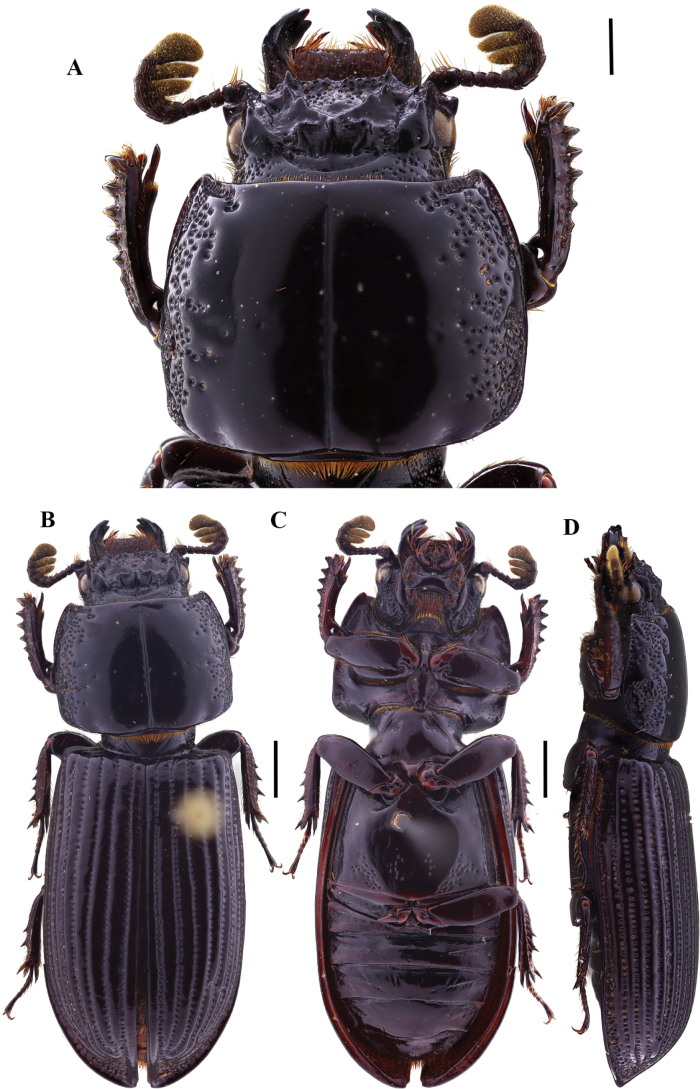
*Rhodocanthopusmaillei* (Percheron, 1841) **A** head and pronotum in dorsal view **B** habitus dorsal **C** habitus ventral **D** habitus lateral. Scale bars: 1.0 mm (**A**); 2.0 mm (**B, C, D**).

###### Comments.

Species very similar to *R.maillei*, from which it differs by its larger size, reduce punctation on the pronotum, which do not reach the mid zone and the metasternal disc not abundantly punctate.

##### ﻿15. Rhodocanthopus rufiventris

Taxon classificationAnimaliaColeopteraPassalidae

(Jiménez-Ferbans, Reyes-Castillo & González, 2016)

3BDEE053-E734-53D8-BECF-DCC3D809C8A4

Fig. 18

###### Diagnosis.

14.0–16.5 mm total length. Body flattened. Anterior border of frons with strong middle indentation, on the sides of which are two insinuated tubercles. Mediofrontal and laterofrontal fused, large. Central tubercle with apex not free. Lateroposterior tubercles distinct and transverse. Eyes reduced. Antennal club tri-lamellate, lamellae short. Lacinia with apex bidentate. Mediobasal area of mentum protruding and glabrous. Marginal groove widened, occupying 1/2 of the anterior margin of the pronotum. Prosternellum rhomboidal, truncate. Mesosternum glabrous; mesosternal scars elongated and conspicuous. Metasternum glabrous anterolaterally and in lateral groove; disc smooth and delimited by punctations posteriorly to laterally. Humeri glabrous; epipleura with scattered setae in basal area. Anterior ventral border of the profemur with conspicuous groove. Meso- and metatibiae with strong spines.

**Figure 18.**
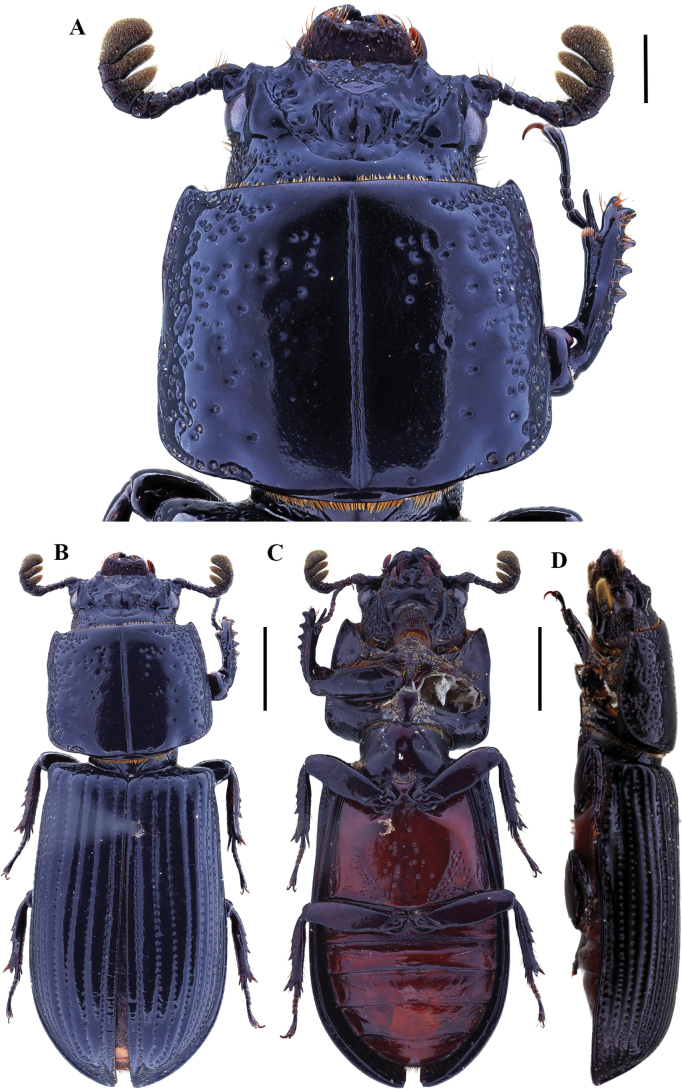
*Rhodocanthopusrufiventris* (Jiménez-Ferbans, Reyes-Castillo & González, 2016) **A** head and pronotum in dorsal view **B** habitus dorsal **C** habitus ventral **D** habitus lateral. Scale bars: 1.0 mm (**A**); 2.0 mm (**B, C, D**).

###### Comments.

Described from the Chocó department of Colombia (Jiménez-Ferbans et al. 2016), this species seems to be endemic to the Chocó-Magdalena province.

##### ﻿16. Spasalus crenatus

Taxon classificationAnimaliaColeopteraPassalidae

(MacLeay, 1819)

72C7D368-F6FB-544E-A86B-E0D325E4BA72

Fig. 19

###### Diagnosis.

17.6–18.2 mm total length. Body robust. Mediofrontal and laterofrontal fused, large. Central tubercle with apex not free. Eyes large. Antennal with five lamellae. Lacinia with apex unidentate. Mediobasal area of mentum protruding, with setae along almost its entire extension. Marginal groove widened, occupying 2/3 of the anterior margin of the pronotum. Anterior border of mentum almost straight. Prosternellum rhomboidal. Anterior ventral border of the profemur with conspicuous groove.

**Figure 19.**
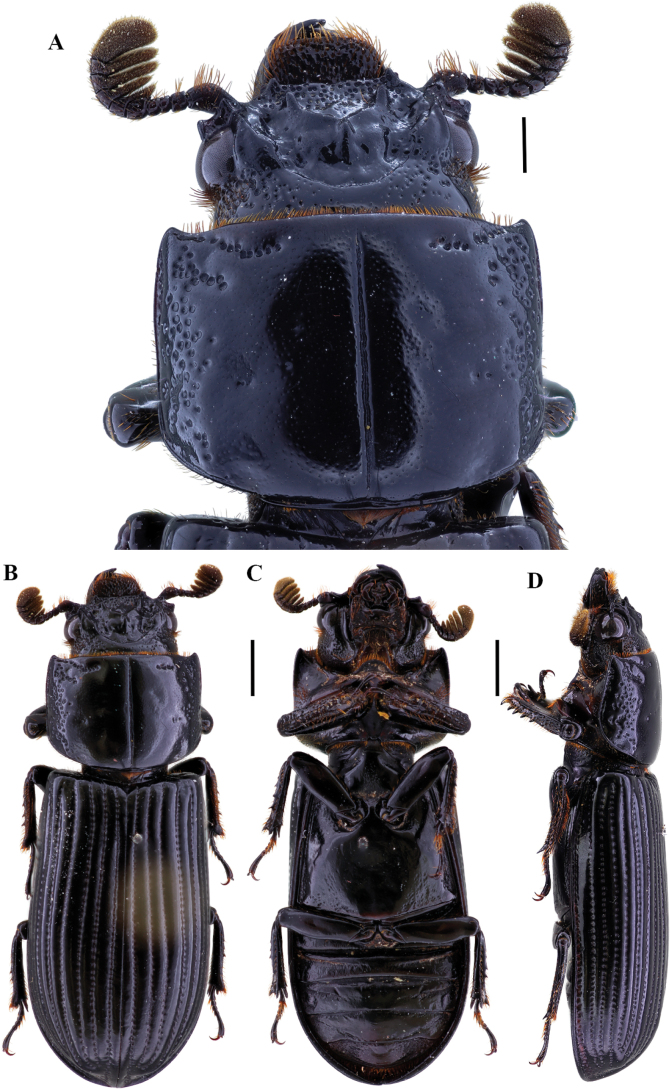
*Spasaluscrenatus* (MacLeay, 1819) **A** head and pronotum in dorsal view **B** habitus dorsal **C** habitus ventral **D** habitus lateral. Scale bars: 1.0 mm (**A**); 2.0 mm (**B, C, D**).

###### Comments.

Distributed widely in South America, this species has been recorded also in Lesser Antilles and Puerto Rico (Jiménez-Ferbans et al. 2015).

##### ﻿17. Spasalus paulinae

Taxon classificationAnimaliaColeopteraPassalidae

Amat-García & Fonseca, 1998

AF01CC46-086E-5C0E-9B24-FBA12D2792B1

###### Diagnosis.

15.75–16.30 mm total length. Body robust. Mediofrontal and laterofrontal fused, large. Central tubercle with apex not free. Eyes large. Antennal with five lamellae. Lacinia with apex unidentate. Mediobasal area of mentum protruding, without setae in the posterior border. Marginal groove widened, occupying 2/3 of the anterior margin of the pronotum. Anterior border of mentum almost straight. Prosternellum rhomboidal. Anterior ventral border of the profemur with conspicuous groove.

Comments. Known only from material type from Sierra Nevada de Santa Marta. The differences between *S.crenatus* and *S.paulinae* are subtle, they may be synonymous.

#### Tribe Proculini

The species of this tribe are recognized by having a frontoclypeus, below which the clypeus is hidden (Boucher 2006).

##### ﻿18. Heliscus eclipticus

Taxon classificationAnimaliaColeopteraPassalidae

(Truqui, 1857)

9EB41432-C97C-5246-AA2B-1D9CABC9EBC9

Fig. 20

###### Diagnosis.

28.7–32.5 mm total length. Anterior border of the labrum straight or slightly concave. Frontoclypeus straight, not swollen in the middle. Frontal-clypeal suture present and strong. Internal tubercles conspicuous, joined to central tubercle by Y-shaped ridges. Central tubercle small, with apex not free; lateroposterior tubercles distinct and transverse, with a superior groove extending over the total length of the tubercles. Frontal fossae pubescent posteriorly. Postfrontal groove complete, slightly erased in the middle. Mediobasal area of the mentum glabrous. Marginal groove over anterior border of the pronotum not expanded. Mesosternum glabrous. Metasternum pubescent anterolaterally and in lateral groove; disc not delimited by punctations. Mesotibiae and metatibiae with a small spine or unarmed. Humeri and epipleura glabrous; anterior vertical face of elytra pubescent.

**Figure 20.**
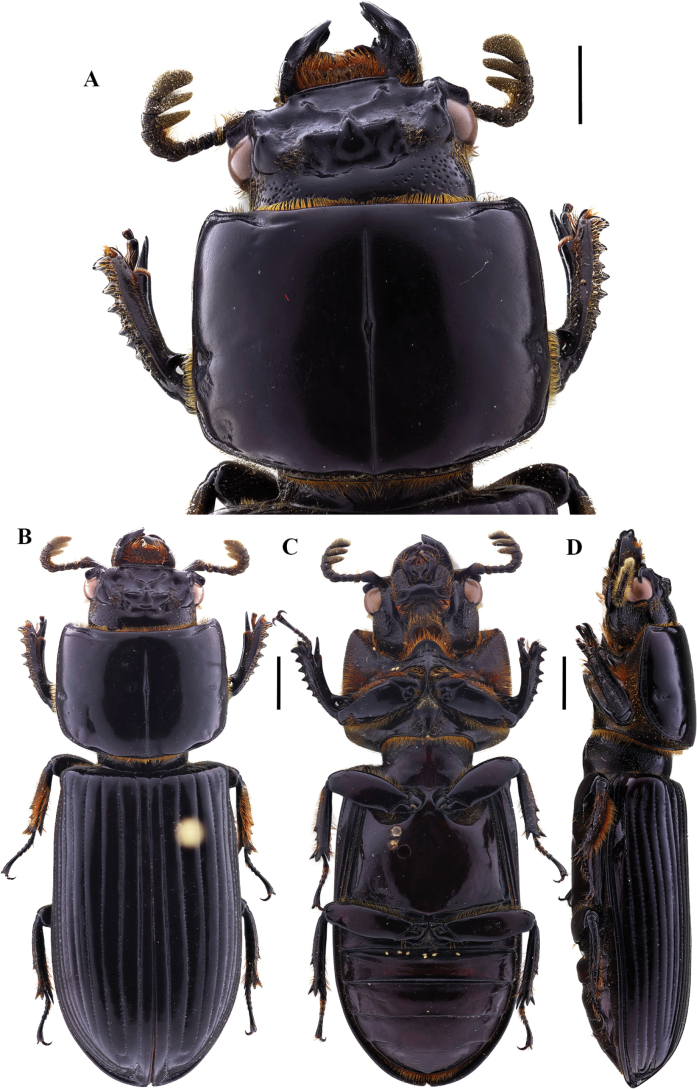
*Heliscuseclipticus* (Truqui, 1857) **A** head and pronotum in dorsal view **B** habitus dorsal **C** habitus ventral **D** habitus lateral. Scale bars: 2.0 mm (**A**); 3.0 mm (**B, C, D**).

##### ﻿19. Odontotaenius striatopunctatus

Taxon classificationAnimaliaColeopteraPassalidae

(Percheron, 1835)

5716BA15-245B-5D6D-BF82-90A35D624F16

Fig. 21

###### Diagnosis.

28.8 mm total length. Anterior border of the labrum straight or slightly concave. Frontoclypeus swollen in the middle. Frontal-clypeal suture present and strong. Internal tubercles small, not joined to the central tubercle. Central tubercle large, with apex free; lateroposterior tubercles absent. Frontal fossae glabrous. Postfrontal groove complete. Mediobasal area of mentum glabrous. Marginal groove over anterior border of pronotum not expanded. Mesosternum glabrous. Metasternum with scarce pubescence anterolaterally and in lateral groove; disc delimited by punctations posteriorly. Mesotibiae and metatibiae with a small spine. Humeri and epipleura glabrous; anterior vertical face of elytra pubescent.

**Figure 21.**
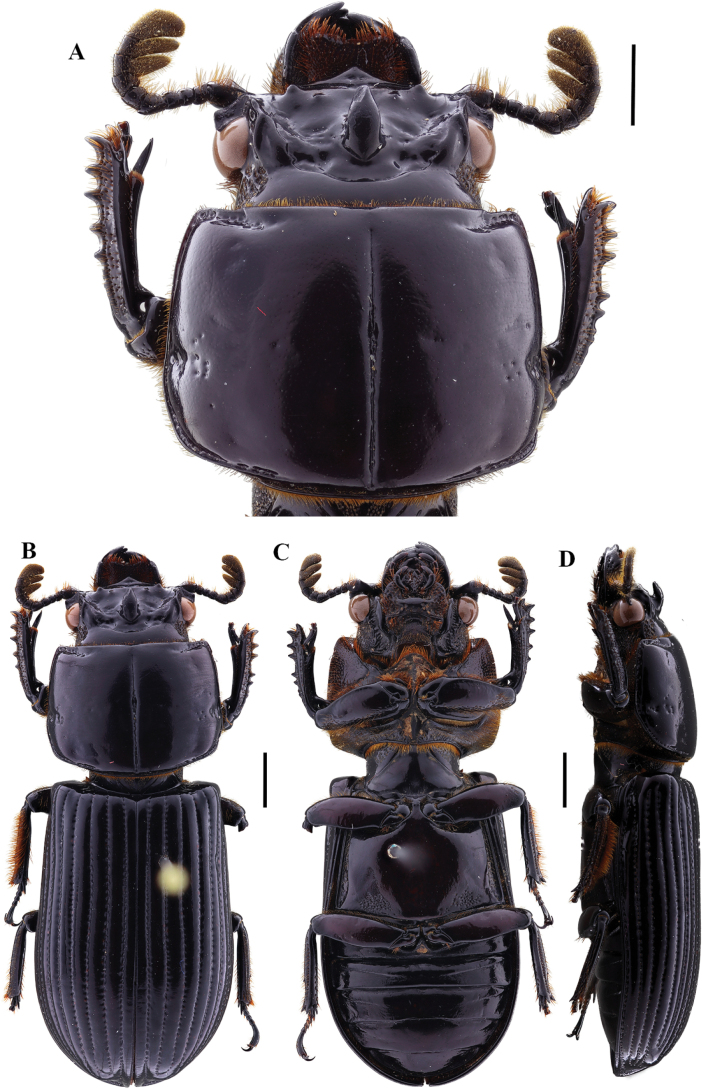
*Odontotaeniusstriatopunctatus* (Percheron, 1835) **A** head and pronotum in dorsal view **B** habitus dorsal **C** habitus ventral **D** habitus lateral. Scale bars: 2.0 mm (**A**); 3.0 mm (**B, C, D**).

##### ﻿20. Popilius erotylus

Taxon classificationAnimaliaColeopteraPassalidae

Reyes-Castillo & Castillo, 1992

94756784-8527-5040-AEE5-A9B9C423C473

Fig. 22

###### Diagnosis.

23.1–25.0 mm total length. Anterior border of the labrum slightly concave. Frontoclypeus straight or slightly concave. Frontal-clypeal suture present and strong. Internal tubercles conspicuous, joined to central tubercle by Y-shaped ridges. Central tubercle small, with apex not free and posterior to level of lateroposterior tubercles which are conspicuous and transverse. Frontal fossae pubescent. Postfrontal groove complete. Mediobasal area of the mentum glabrous. Marginal groove over anterior border of the pronotum slightly expanded. Mesosternum glabrous. Metasternum with scarce pubescence anterolaterally and in lateral groove; disc not delimited by punctations. Meso- and metatibiae with small spines. Humeri with scarce setae; epipleura glabrous; anterior vertical face of elytra pubescent.

**Figure 22.**
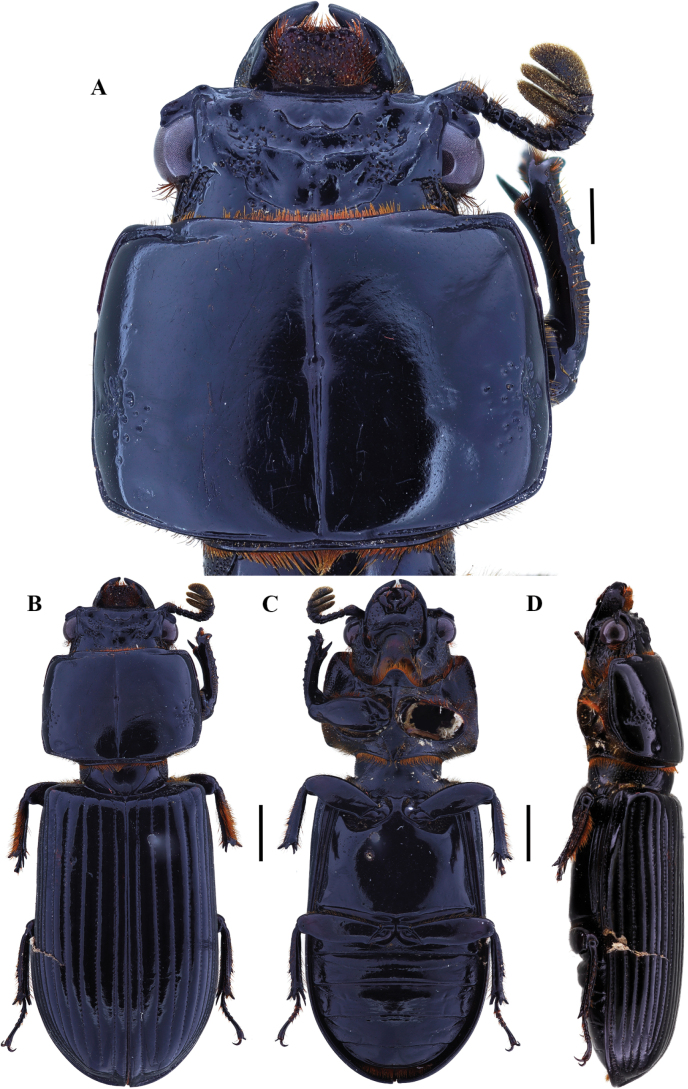
*Popiliuserotylus* Reyes-Castillo & Castillo, 1992 **A** head and pronotum in dorsal view **B** habitus dorsal **C** habitus ventral **D** habitus lateral. Scale bars: 1.0 mm (**A**); 2.0 mm (**B, C, D**).

###### Comments.

*Popiliuserotylus* was described from Panama. The specimens studied here show some setal variation in comparison with the type material; they could be a new species very closely related to *P.erotylus*.

##### ﻿21. Popilius gibbosus

Taxon classificationAnimaliaColeopteraPassalidae

(Burmeister, 1847)

915F3209-943B-5366-8EE7-2CA9A2962ACD

Fig. 23

###### Diagnosis.

21.4–23.5 mm total length. Anterior border of the labrum slightly concave. Frontoclypeus straight or slightly expanded forward. Frontal-clypeal suture present and strong. Internal tubercles small, joined to central tubercle by Y-shaped ridges. Central tubercle small, with apex not free and almost even with lateroposterior tubercles which are distinct and transverse. Frontal fossae pubescent. Postfrontal groove complete. Mediobasal area of the mentum glabrous. Marginal groove over anterior border of the pronotum not expanded. Mesosternum glabrous. Metasternum pubescent anterolaterally and in lateral groove, pubescence extending beyond lateral groove; disc not delimited by punctations. Meso- and metatibiae with a small spine. Humeri and epipleura glabrous or with scarce setae basally; anterior vertical face of elytra pubescent.

**Figure 23.**
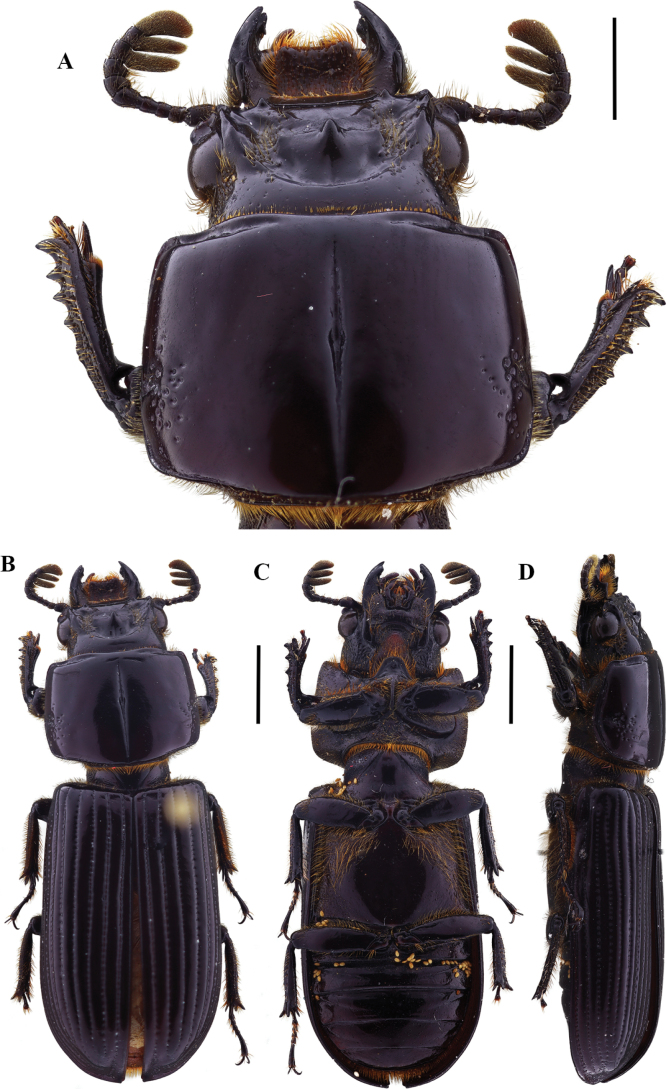
*Popiliusgibbosus* (Burmeister, 1847) **A** head and pronotum in dorsal view **B** habitus dorsal **C** habitus ventral **D** lateral view. Scale bars: 2.0 mm (**A**); 3.0 mm (**B, C, D**).

###### Comments.

Common species from the north of the Eastern Cordillera (Serranía del Perijá and Sierra de Venezuela) (Gillogly 2005). It is found in the intermediate and high areas of the Andes, where it is endemic.

##### ﻿22. Popilius marginatus

Taxon classificationAnimaliaColeopteraPassalidae

(Percheron, 1835)

CE7B01B0-6969-5ADE-928C-D1E6C2E2570F

Fig. 24

###### Diagnosis.

20.6–24.4 mm total length. Anterior border of the labrum slightly concave. Frontoclypeus straight or slightly concave. Frontal-clypeal suture present and strong. Internal tubercles conspicuous, joined to central tubercle by Y-shaped ridges. Central tubercle small, with apex not free and even with lateroposterior tubercles which are conspicuous and transverse. Frontal fossae glabrous or rarely pubescent. Postfrontal groove complete. Mediobasal area of the mentum glabrous. Marginal groove over anterior border of the pronotum slightly expanded. Mesosternum glabrous. Metasternum with scarce pubescence anterolaterally and in lateral groove; disc not delimited by punctations. Meso- and metatibiae with small spines. Humeri with scarce setae; epipleura glabrous; anterior vertical face of elytra pubescent.

**Figure 24.**
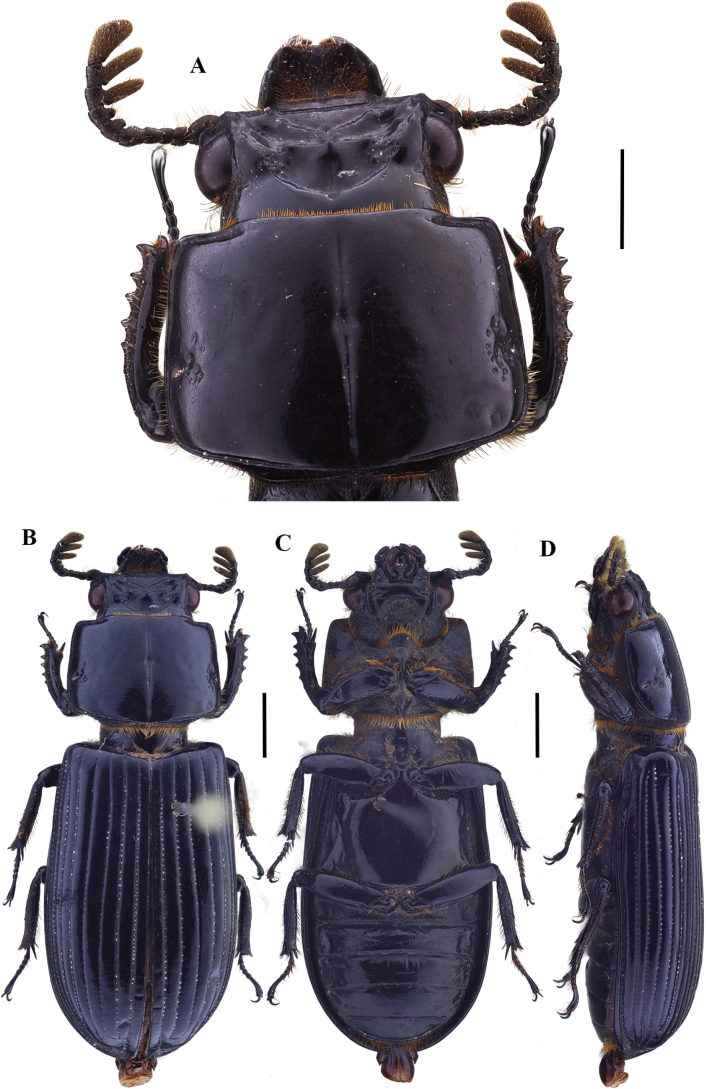
*Popiliusmarginatus* (Percheron, 1835) **A** head and pronotum in dorsal view **B** habitus dorsal **C** habitus ventral **D** lateral view. Scale bars: 2.0 mm (**A**); 3.0 mm (**B, C, D**).

###### Comments.

*P.marginatus* has been recorded from Argentina, Bolivia, Brazil, Colombia, French Guiana, Peru and Suriname (Gillogly 2005; Jiménez-Ferbans et al. 2013). Some specimens from SNSM have some differences with the characteristics described by Gillogly (2005) and could be a new species.

##### ﻿23. Verres corticicola

Taxon classificationAnimaliaColeopteraPassalidae

Kaup, 1871

4EF0FFE9-0C8E-586A-A66E-2E6052E1A307

Fig. 25

###### Diagnosis.

32.9 mm total length. Anterior border of the labrum deeply concave, with an excavation in labrum behind concavity of margin. Frontoclypeus slightly curved, with a central notch. Frontal-clypeal suture absent. Internal tubercles large, with free apex projecting forward, not joined to central tubercle. Central tubercle short, with apex free projected forward; lateroposterior tubercles conspicuous and transverse. Frontal fossae glabrous. Postfrontal groove complete. Mediobasal area of the mentum glabrous. Marginal groove over anterior border of the pronotum expanded. Mesosternum glabrous. Metasternum pubescent anterolaterally and in lateral groove, pubescence extending beyond lateral groove; disc delimited by punctations posteriorly. Meso- and metatibiae unarmed. Humeri with scarce setae; epipleura glabrous; anterior vertical face of elytra glabrous.

**Figure 25.**
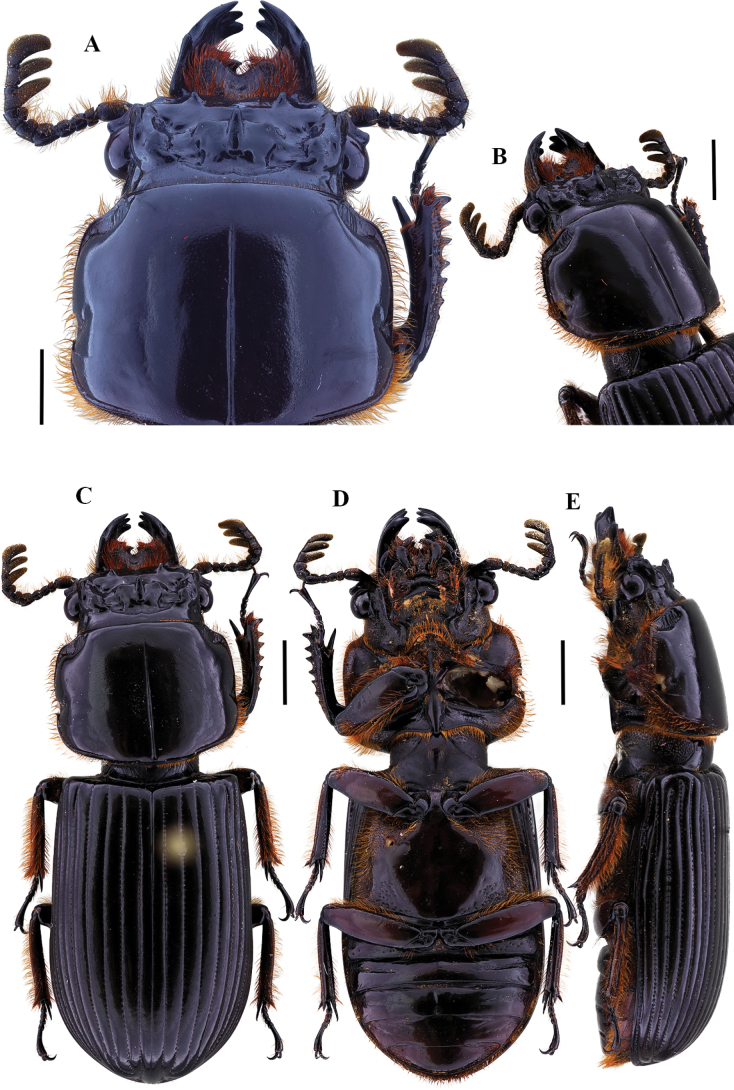
*Verrescorticicola* Kaup, 1871 **A** head and pronotum in dorsal view **B** head and pronotum in dorso-lateral view **C** habitus dorsal **D** habitus ventral **E** habitus lateral. Scale bars: 2.0 mm (**A, B**); 3.0 mm (**C, D, E**).

###### Comments.

Marshall (2000) cited this species from Mexico to Costa Rica, indicating that it is common to collect. In the Colombian Caribbean it seems to be relegated to the humid forests of the biogeographical province of Chocó-Magdalena.

##### ﻿24. Verres hageni

Taxon classificationAnimaliaColeopteraPassalidae

Kaup, 1871

10DF852B-8D84-58FE-9C5B-C13A1EE4FA58

Fig. 26

###### Diagnosis.

34.4–38.5 mm total length. Anterior border of the labrum deeply concave, with an excavation in labrum behind concavity of margin. Frontoclypeus slightly curved, with a central notch. Frontal-clypeal suture absent. Internal tubercles small, blunt, not joined to central tubercle. Central tubercle with apex slightly free, oblique; lateroposterior tubercles distinct and transverse. Frontal fossae glabrous. Postfrontal groove complete. Mediobasal area of the mentum glabrous. Marginal groove over anterior border of the pronotum not expanded. Mesosternum glabrous. Metasternum pubescent anterolaterally and in lateral groove, pubescence extending beyond lateral groove; disc delimited by punctations posteriorly. Meso- and metatibiae unarmed. Humeri with scarce setae; epipleura glabrous; anterior vertical face of elytra glabrous.

**Figure 26.**
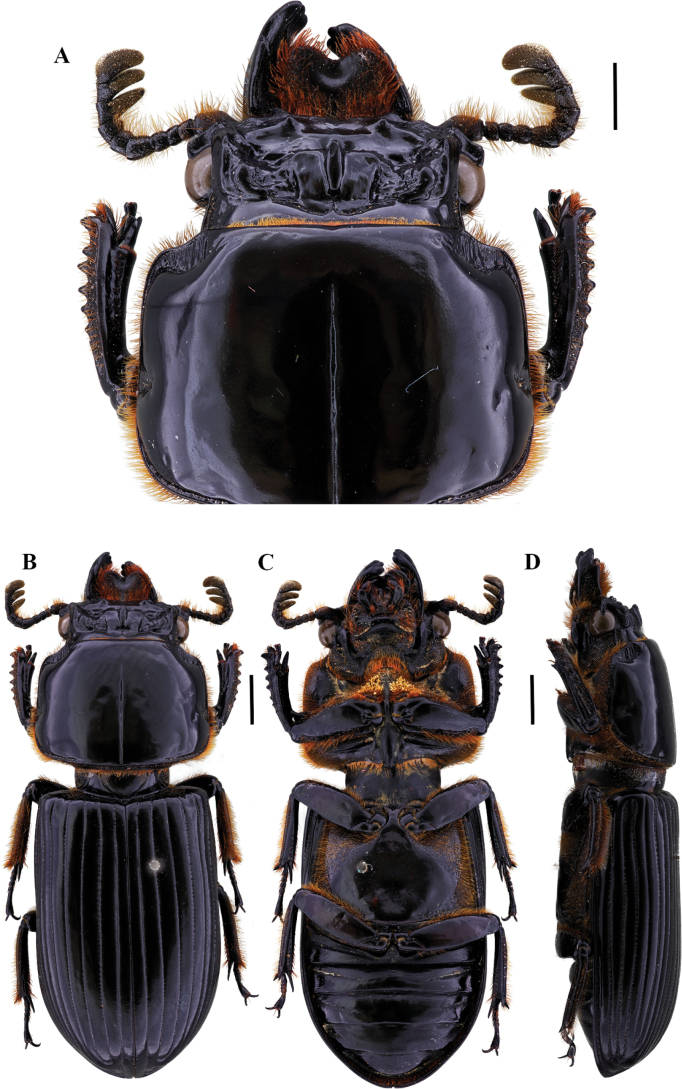
*Verreshageni* Kaup, 1871 **A** head and pronotum in dorsal view **B** habitus dorsal **C** habitus ventral **D** lateral view. Scale bars: 2.0 mm (**A**); 3.0 mm (**B, C, D**).

###### Comments.

Jiménez-Ferbans and Amat-García (2009) cited *V.hageni* from the Caribbean coast of Colombia, but without citing specimens. Consequently, specimens examined in this work are the first from the region.

##### ﻿25. Veturius (Ouayana) cirratus

Taxon classificationAnimaliaColeopteraPassalidae

Bates, 1886

E9DFCBAF-50AF-5FB3-A990-D83D76A632EA

Fig. 27

###### Diagnosis.

28.7–33.6 mm total length. Anterior border of the labrum slightly concave. Frontoclypeus straight. Frontal-clypeal suture absent. Internal tubercles small, joined to central tubercle by V-shaped ridges. Central tubercle short, with apex not free; lateroposterior tubercles large, conspicuous, and transverse. Frontal fossae pubescent. Postfrontal groove incomplete, interrupted behind central tubercle. Mediobasal area of the mentum pubescent. Marginal groove over anterior border of the pronotum not expanded. Mesosternum with two rows of setae running longitudinally parallel. Metasternum pubescent anterolaterally and in lateral groove; disc not delimited by punctations. Meso- and metatibiae unarmed. Humeri and epipleura glabrous; anterior vertical face of elytra pubescent.

**Figure 27.**
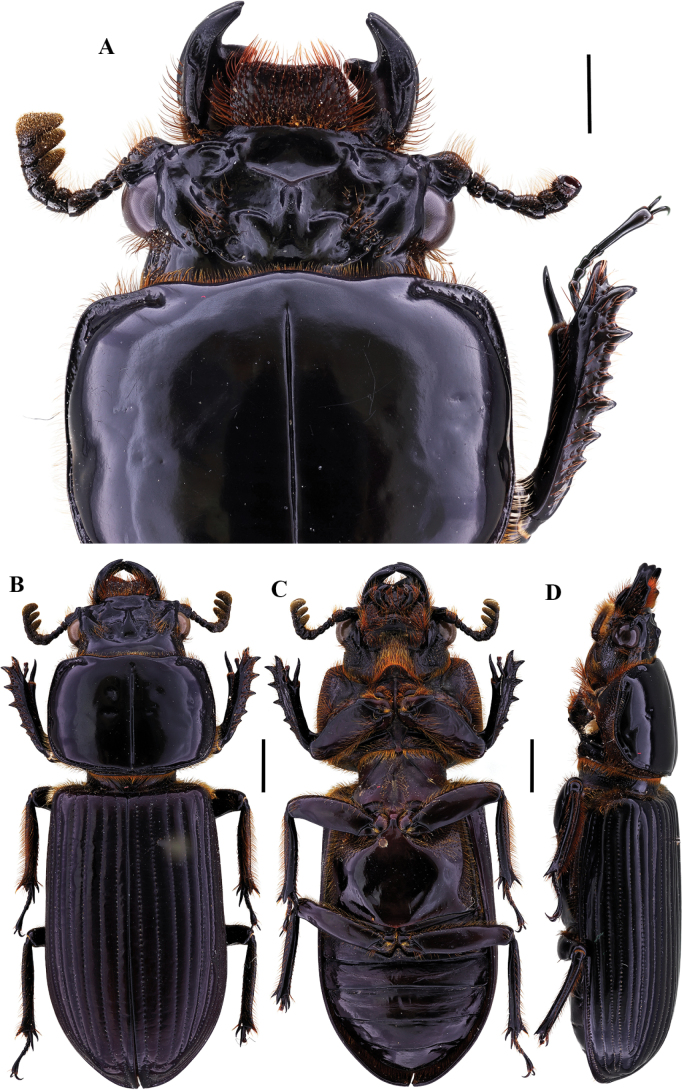
Veturius (Ouayana) cirratus Bates, 1886 **A** head and pronotum in dorsal view **B** habitus dorsal **C** habitus ventral **D** habitus lateral. Scale bars: 2.0 mm (**A**); 3.0 mm (**B, C, D**).

##### ﻿26. Veturius (Publius) impressus

Taxon classificationAnimaliaColeopteraPassalidae

Hincks, 1852

256A05BD-D431-5F51-8E76-7D7071B2775A

Fig. 28

###### Diagnosis.

40.6–47.9 mm total length. Anterior border of the labrum concave. Frontoclypeus straight. Frontal-clypeal suture absent. Internal tubercles prominent, joined to central tubercle by V-shaped ridges. Central tubercle large, with apex not free; lateroposterior tubercles absent. Frontal fossae glabrous. Postfrontal groove complete. Mediobasal area of the mentum glabrous. Marginal groove over anterior border of the pronotum not expanded. Mesosternum with scarce setae. Metasternum pubescent anterolaterally and in lateral groove; disc not delimited by punctations. Meso- and metatibiae unarmed. Humeri with scarce setae basally; epipleura glabrous; anterior vertical face of elytra sparsely pubescent.

**Figure 28.**
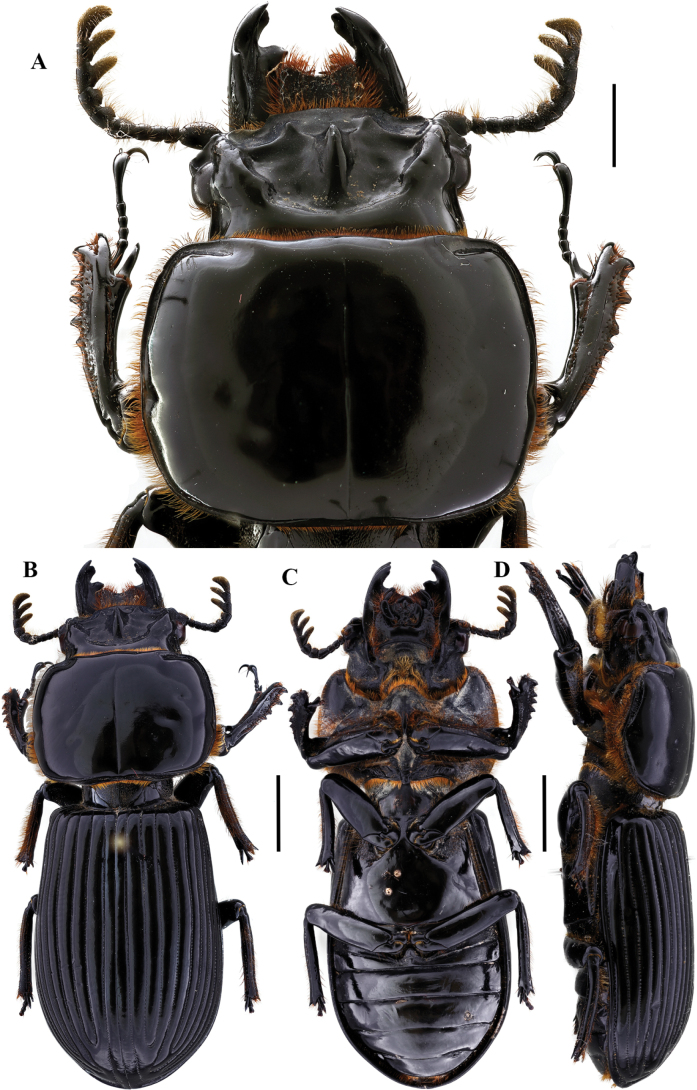
Veturius (Publius) impressus Hincks, 1852 **A** head and pronotum in dorsal view **B** habitus dorsal **C** habitus ventral **D** lateral view. Scale bars: 4.0 mm (**A**); 5.0 mm (**B, C, D**).

###### Comments.

This species seems to be common in the middle elevations of Sierra Nevada de Santa Marta. It has been collected in both forest areas and in shady coffee plantations; in the latter within logs of *Erythrina* sp. (Leguminosaeae), in which there were also colonies of *Leptogenys* sp. (Formicidae). *Veturiusimpressus* is the only Proculini endemic to the Caribbean region of Colombia.

##### ﻿27. Veturius (Veturius) aspina

Taxon classificationAnimaliaColeopteraPassalidae

Kuwert, 1898

4BADAAC1-F0B1-5398-90C0-58555D9A877F

Fig. 29

###### Diagnosis.

47.5–50.4 mm total length. Anterior border of the labrum straight or slightly concave. Frontoclypeus almost straight. Frontal-clypeal suture absent. Internal tubercles small, joined to central tubercle by V-shaped ridges. Central tubercle small, with apex slightly free, upward projected; lateroposterior tubercles large, conspicuous, and transverse. Frontal fossae glabrous. Postfrontal groove complete. Mediobasal area of the mentum pubescent. Marginal groove over anterior border of the pronotum expanded. Mesosternum glabrous. Metasternum pubescent anterolaterally and in lateral groove; disc not delimited by punctations. Meso- and metatibiae unarmed. Humeri and epipleura glabrous; anterior vertical face of elytra pubescent.

**Figure 29.**
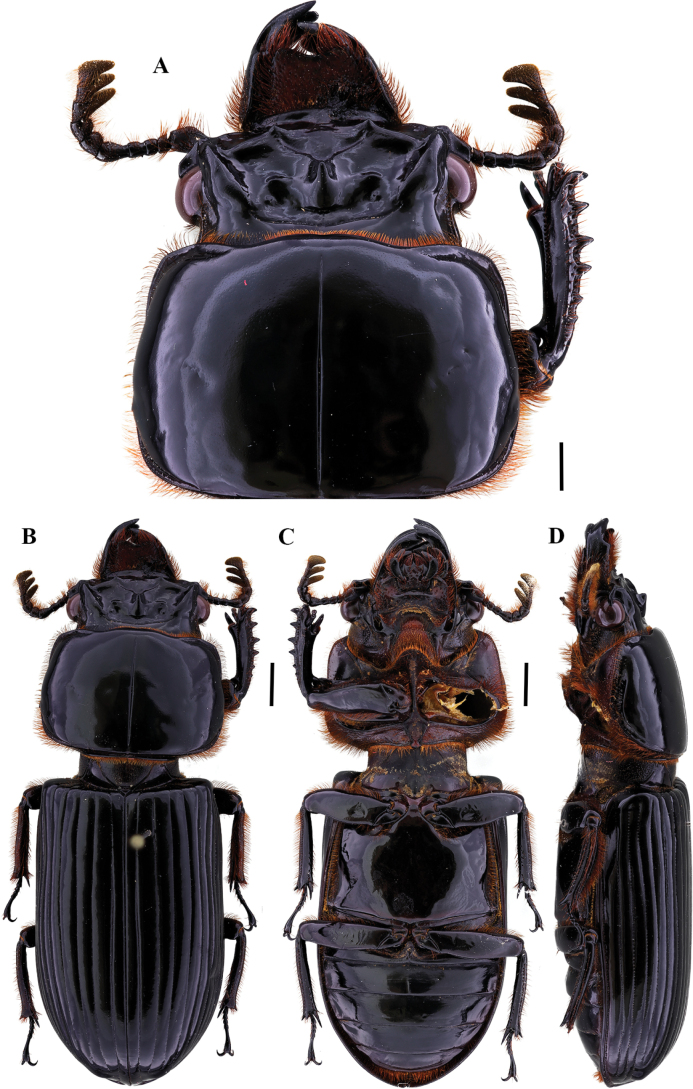
Veturius (Veturius) aspina Kuwert, 1898 **A** head and pronotum in dorsal view **B** habitus dorsal **C** habitus ventral **D** lateral view. Scale bars: 2.0 mm (**A**); 4.0 mm (**B, C, D**).

##### ﻿28. Veturius (Veturius) standfussi

Taxon classificationAnimaliaColeopteraPassalidae

Kuwert, 1891

7E0C231C-2EF2-5E98-A354-AC70EDEDD13B

Fig. 30

###### Diagnosis.

41.4–46.5 mm total length. Anterior border of the labrum straight or slightly concave. Frontoclypeus almost straight. Frontal-clypeal suture absent. Internal tubercles small, joined to central tubercle by V-shaped ridges. Central tubercle small, with apex not free; lateroposterior tubercles large, distinct, and transverse. Frontal fossae with some setae. Postfrontal groove complete. Mediobasal area of the mentum glabrous. Marginal groove over anterior border of the pronotum expanded. Mesosternum glabrous. Metasternum pubescent anterolaterally and in lateral groove; disc not delimited by punctations. Meso- and metatibiae with a small spine. Humeri and epipleura glabrous; anterior vertical face of elytra pubescent.

**Figure 30.**
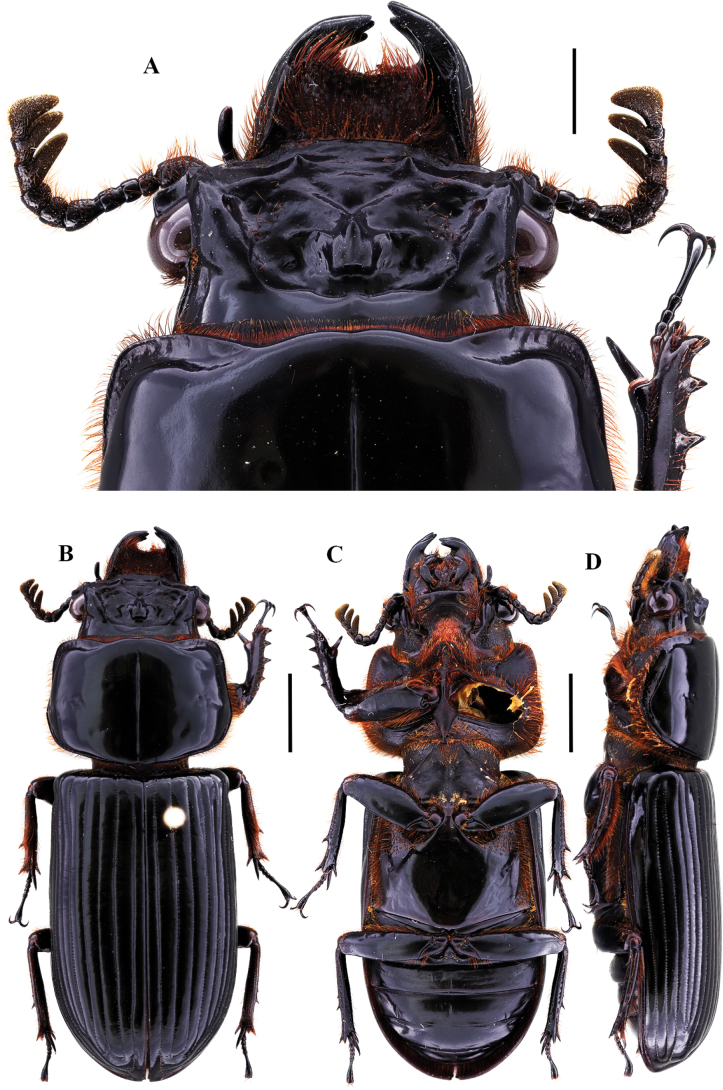
Veturius (Veturius) standfussi Kuwert, 1891 **A** head and pronotum in dorsal view **B** habitus dorsal **C** habitus ventral **D** lateral view. Scale bars: 4.0 mm (**A**); 5.0 mm (**B, C, D**).

###### Comments.

Originally described from Venezuela, this species is distributed in the Andes of Bolivia, Colombia, Ecuador, Peru, and Venezuela (Boucher 2006).

### ﻿Species with doubtful records

Jiménez-Ferbans and Amat-García (2009) cited a specimen of Passalus (Mitrorhinus) arrowi Hincks, 1934 labeled as “Sierra Nevada de Santa Marta, San Pedro de la Sierra, 1530 m., 20 abr 1992, G. Amat leg. (1ej, GAG P-606)”. However, as Jiménez-Ferbans et al. (2018b) pointed out, this species is endemic to the lowlands of the Chocó biogeographic province. Likewise, after many visits to the area, no other specimen of this species has been collected. This leads us to think that the citation may be due to a mislabeled specimen. Consequently, the citation of *P.arrow* for the Sierra Nevada de Santa Marta must be rejected.

### ﻿Key to the Passalidae from the Caribbean Coast of Colombia

(Spanish version available as Suppl. material 3)

**Table:** 

1	Clypeus hidden below frons, with anterior angles below the mediofrontal tubercles (Passalini) (Figs 2–19)	**2**
–	Clypeus (frontoclypeus) exposed dorsally, with anterior angles in front of the border of the frons (Proculini) (Figs 20–28)	**18**
2	Antennal club with 5 lamellae (Figs 16, 19). Maxilla with lacinia unidentate in the apical third	**3**
–	Antennal club with 3 lamellae of similar length (Figs 2, 3) or a fourth (basal, Fig. 4) tomentose lamella distinctly shorter than the others (*Passalusinterstitialis*). Maxilla with lacinia bidentate in the apical third	**5**
3	Prosternellum pentagonal, with posterior apex very wide (Fig. 16C). Femur I without marginal groove on the anterior border of the ventral face (Fig. 16C). Body flattened. (17.5–18.8 mm)	***Paxillusleachi***
–	Prosternellum rhomboidal (Fig. 19C). Femur I with marginal groove on the anterior border of the ventral face. Body robust. (*Spasalus*)	**4**
4	Medial basal mentum almost entirely with punctures and setae. Length 17.6–18.2 mm	***Spasaluscrenatus***
–	Medial basal mentum with punctures and setae only on posterior border. Length 15.75–16.30 mm	***Spasaluspaulinae***
5	Secondary internal tubercles on frontal ridges present (Fig. 17A). Eyes reduced. Meso- and metatibiae with strong spines on the external edge (Fig. 17B). Small size, 14.0–19.8 mm. (*Rhodocanthopus*)	**6**
–	Secondary internal tubercles on frontal ridges absent (Fig. 3A). Eyes not reduced. Meso- and metatibiae without strong spines on the external edge (Fig. 3B). Variable size, usually > 30 mm. (*Passalus*)	**7**
6	Body robust. Abdominal tergites black in color in mature adults. Length 16.3–19.8 mm	***Rhodocanthopusmaillei***
–	Body flattened. Abdominal tergites reddish in color (even in mature adults). Length 14.0–16.5 mm	***Rhodocanthopusrufiventris***
7	Anterior border of frons with 2 secondary mediofrontal tubercles (Fig. 4A). If border straight, then central tubercle with apex distinctly free (Fig. 7A). (Subgenus Passalus)	**8**
–	Anterior border of frons straight or almost straight, without secondary mediofrontal tubercles. Central tubercle always with apex not free (Figs 12A, 14A). (Subgenus Pertinax)	**14**
8	Central tubercle with apex very free, reaching or surpassing the anterior border of the frons (Figs 6A, 9A)	**9**
–	Central tubercle with apex not free or slightly free, not reaching the anterior border of the frons (Figs 2D, 3D, 4A)	**11**
9	Body svelte and flattened. Macropterous (Fig. 6D). Humeri fully pubescent (Fig. 6B). Length 28.7–34.2 mm	***Passalusserankuai***
–	Body robust, subcylindrical (Figs 7D, 9D). Hemi- or Braquipterous. Humeri pubescence basally	**10**
10	Anterior frontal edge without middle indentation or secondary mediofrontal tubercles (Figs 7A, 8C). Central tubercle without a sulcus in the posterior part (Fig. 7A). Prosternellum without longitudinal groove (Fig. 8B). Humeri pubescent (Fig. 7D). Length 30.1 mm	***Passaluschechai* sp. nov.**
–	Anterior frontal edge with middle indentation and rudimentary secondary mediofrontal tubercles (Fig. 10C). Central tubercle with a sulcus in the posterior part (Figs 9A, 10C). Prosternellum with longitudinal groove (Fig. 10B). Humeri with scarce pubescence basally (Fig. 9D). Length 36.7–37.8 mm	***Passalusflorezi* sp. nov.**
11	Body flattened. Central tubercle with apex not free (Fig. 4D). Antennal club with 4 lamellae, the fourth very reduced and tomentose (Fig. 4A). Length 23.6–29.9 mm	***Passalusinterstitialis***
–	Body robust. Central tubercle with apex slightly free (Fig. 5D). Antennal club with only 3 lamellae (Fig. 5A)	**12**
12	Central tubercle distinctly free but short (Fig. 2A, D). Mesosternum with abundant pubescence, extending beyond mesosternal scars (Fig. 2C). Length 39.6–46.5 mm	***Passalusconiferus***
–	Central tubercle barely free (Fig. 5D). Mesosternum glabrous or with scarce setae on mesosternal scars (Figs 4C, 5C)	**13**
13	Last abdominal sternite with medially incomplete groove (Fig. 3C). Body long, 41.2–52.7 mm	***Passalusinterruptus***
–	Last abdominal sternite with medially complete groove (Fig. 5C). Body length medium to long, 28.4–41.7 mm	***Passaluspunctiger***
14	Humeri and epipleura with dense pubescence (Fig. 14C, D). Length 20.1–23.2 mm	***Passalusrugosus***
–	Humeri and epipleura glabrous or with a few setae at the base (Figs 12C, D, 15C, D)	**15**
15	Frontal fossae with sparse long setae (Fig. 12A). Length 22.9–25.5 mm	***Passaluspaucuvillosus***
–	Frontal fossae glabrous (Fig. 13A)	**16**
16	Eyes reduced (Fig. 11A). Hemibrachypterous. Mesosternum without mesosternal scars, indicated only by an opaque area (Fig. 11C). Body long. Length 31.6–34.2 mm	***Passalusgaboi***
–	Eyes not reduced (Figs 13A, 15A). Macropterous. Mesosternum with strong mesosternal scars (Figs 13C, 15C). Body small to medium, 19.5–26.0 mm	**17**
17	Internal tubercles large, with free apex (Fig. 15A). Humeri with long setae at the very base (Fig. 15C). Length 19.5–22.4 mm	***Passalusunimagdalenae***
–	Internal tubercles small, with apex not free (Fig. 13A). Humeri glabrous (Fig. 13C, D). Length 21.8–26.0 mm	***Passaluspunctatostriatus***
18	Clypeus swollen anteromedially (Fig. 21A). Central tubercle large, with apex free (Fig. 21D). Length 28.8 mm	***Odontotaeniusstriatopunctatus***
–	Clypeus not swollen anteromedially (Figs 20A, 22A). Central tubercle small with apex slightly free or not free (Figs 20A, D, 22A, D)	**19**
19	Anterior labral border deeply concave, with an excavation behind concavity (dorsal depression sensu Marshall 2000) (Figs 25A, 26A) (*Verres*)	**20**
–	Anterior labral border straight or slightly concave or convex, without an excavation behind border (Figs 24A, 27A)	**21**
20	Internal tubercles large, with free apex projecting forwards, surpassing the frontoclypeus (Fig. 25A, B). Mesosternum dull (Fig. 25D). Length 32.9 mm	***Verrescorticicola***
–	Internal tubercles small and blunt, not surpassing the frontoclypeus (Fig. 26A). Mesosternum shiny (Fig. 26C). Length 34.4–38.5 mm	***Verreshageni***
21	Frontoclypeal suture strong (Figs 20A, 24A). Anterior border of pronotum straight (Figs 20A, 24A, B)	**22**
–	Frontoclypeal suture absent (Figs 27A, 28A). Anterior border of pronotum heavily sinuous (Fig. 30A, B) (*Veturius*)	**25**
22	Metasternal pubescence restricted to the mesocoxal cavity and lateral fossa (Fig. 20C). Median last abdominal sternite rugose and tuberculate (Fig. 20C). Length 28.7–32.5 mm	***Heliscuseclipticus***
–	Metasternal pubescence absent (*Popiliuserotylus*, Fig. 22C) or restricted to the mesocoxal and anterior part of lateral fossa (*Popiliusmarginatus*, Fig. 24C) or extended far beyond the mesocoxal cavity and lateral fossa (*Popiliusgibbosus* Fig. 23C). Median last abdominal sternite smooth and not tuberculate (Figs 23C, 24C)	**23**
23	Metasternal pubescence extend way beyond the mesocoxal cavity and lateral groove (Fig. 23C). Length 21.4–23.5 mm	***Popiliusgibbosus***
–	Metasternal pubescence absent or scarce (Figs 22C, 24C)	**24**
24	Tip of central tubercle even with parietal tubercles (Fig. 24A, B). Length 20.6–24.4 mm	***Popiliusmarginatus***
–	Tip of central tubercle posterior to level of parietal tubercles (Fig. 22A). Length 23.1–25.0 mm	***Popiliuserotylus***
25	Lateroposterior tubercles absent (Fig. 28A). Posterior metasternal lateral fossa less wide than mesotibia (Fig. 28C). Brachypterous. Length 40.6–47.9 mm	***Veturiusimpressus***
–	Lateroposterior tubercles present (Figs 27A, 29A). Posterior metasternal lateral fossa at least same width as mesotibia (Figs 27C, 29C). Macropterous	**26**
26	Frontal fossae pubescent, pubescence extending over supraorbital ridges (Fig. 27A). Mesosternum pubescent (Fig. 27C). Elytra opalescent (Fig. 27B). Length 28.7–33.6 mm	***Veturiuscirratus***
–	Frontal fossae glabrous (Fig. 29A) or with scarce setae (Fig. 30A), never extending over supraorbital ridges. Mesosternum glabrous posteriorly (Figs 29C, 30C). Elytra not opalescent (Figs 29B, 30B)	**27**
27	Frontal fossae glabrous (Fig. 29A). Mesotibiae and metatibiae without lateral spines (Fig. 29B, C). Length 47.5–50.4 mm	***Veturiusaspina***
–	Frontal fossae with scarce setae (Fig. 30A). Mesotibiae and metatibiae with small lateral spines (Fig. 30B, C). Length 41.4–46.5 mm	***Veturiusstandfussi***

## ﻿Discussion

With 28 species, the Colombian Caribbean hosts an important richness of passalid species, which also exhibits a unique composition. The dry plain, characteristic of the lowlands of northern Colombia, is dominated by widely distributed species such as *Passaluspunctiger* and *P.interstitialis*, and to a lesser extent by *P.interruptus*. Meanwhile, the mountainous systems provide elements of more restricted distribution, some of them endemic to the Colombian Caribbean, similar to other regions in the Neotropics (e.g., Guatemala and Costa Rica; Beza-Beza et al. pers. comm.; Jiménez-Ferbans et al. 2017). Thus, the Sierra Nevada de Santa Marta (SNSM) monopolizes the elements endemic to the Caribbean region of Colombia, all of them distributed between 1500 and 2100 masl. Of the 14 species recorded in the SNSM, four are exclusive to this region (3 Passalini and 1 Proculini), which highlights the biogeographic importance of this mountain massif. For its part, the Perijá mountain range represents another site with a significantly high richness of Passalidae in the Caribbean (10 species), most of them represented in medium elevations (1500 m), representing Andean elements. Perijá mountain range belongs to the Norandina province and harbors one species endemic to Colombia (*Passalusrugosus*).

Despite having the smallest number of localities sampled, the Chocó-Magdalena is the richest province in the Caribbean region, with 15 species of Passalidae. It is in this province where species shared between the Colombian Pacific and Mesoamerica are also recorded (e.g., *Heliscuseclipticus*, *Odontotaeniusstriatopunctatus*, and *Passaluspaucuvillosus*). Likewise, according to Jiménez-Ferbans et al. (2018b), the Chocó region is the richest area for Passalidae in Colombia, accounting for 34 species (14 of which are endemic). Presumably, ecological and historical factors make the Chocó-Magdalena province a rich area for Passalidae, since this is the most humid area for Colombia (some localities reach rainfall of up to 13,700 mm; Poveda et al. 2004) and it represents a bridge between Mesoamerica and South America fauna, and Beza-Beza et al. (2021) stated that, in addition to Nuclear Mesoamerica, the Chocó-Darien region is also an important center of origin and diversification for Proculini.

By geopolitical divisions (departments), Córdoba has the highest richness is Córdoba (15 species), due to the high richness recorded in the southern portion of the department, which biogeographically belongs to the Chocó-Magdalena province (Hernández-Camacho et al. 1992). The second most diverse department is Magdalena (14), because of the species from Sierra Nevada de Santa Marta. However, an estimation of the number of species for some departments is still premature, because some of them (Cesar, Bolivar, and Atlántico) have very few or no localities sampled (see map). Thus, although in comparative terms the Colombian Caribbean can be considered to have a good level of sampling, there are still extensive areas to be explored or revisited. Such is the case of La Macuira and Perijá mountain range (La Guajira), San Lucas mountain range (Bolívar), Montes de María (Bolívar and Sucre), and the south part of Córdoba, Sucre, and Bolivar departments. Due to the characteristics of these areas and the high species turnover that is evident among the Caribbean mountain systems, it is likely that these other regions will contribute to new elements (i.e., species, genera) to the general species list of the Colombian Caribbean Passalidae. For instance, more than 30 species have been listed for the Norandina region in Colombia; likely several of these species are present in the San Lucas and Perijá mountains ranges, but there are only 11 sampled localities for Perijá and none for San Lucas. To conclude, we recommend prioritizing future explorations in mountainous environments and in the biogeographic province of Chocó-Magdalena.

## Supplementary Material

XML Treatment for Passalus (Passalus) coniferus

XML Treatment for Passalus (Passalus) interruptus

XML Treatment for Passalus (Passalus) interstitialis

XML Treatment for Passalus (Passalus) punctiger

XML Treatment for Passalus (Passalus) serankuai

XML Treatment for Passalus (Passalus) chechai

XML Treatment for Passalus (Passalus) florezi

XML Treatment for Passalus (Pertinax) gaboi

XML Treatment for Passalus (Pertinax) paucuvillosus

XML Treatment for Passalus (Pertinax) punctatostriatus

XML Treatment for Passalus (Pertinax) rugosus

XML Treatment for Passalus (Pertinax) unimagdalenae

XML Treatment for Paxillus leachi

XML Treatment for Rhodocanthopus maillei

XML Treatment for Rhodocanthopus rufiventris

XML Treatment for Spasalus crenatus

XML Treatment for Spasalus paulinae

XML Treatment for Heliscus eclipticus

XML Treatment for Odontotaenius striatopunctatus

XML Treatment for Popilius erotylus

XML Treatment for Popilius gibbosus

XML Treatment for Popilius marginatus

XML Treatment for Verres corticicola

XML Treatment for Verres hageni

XML Treatment for Veturius (Ouayana) cirratus

XML Treatment for Veturius (Publius) impressus

XML Treatment for Veturius (Veturius) aspina

XML Treatment for Veturius (Veturius) standfussi

## ﻿Additional information

### Author ORCIDs

Larry Jiménez-Ferbans https://orcid.org/0000-0002-5710-2265

Ana Maestre-Guerra https://orcid.org/0000-0002-2046-8036

Evelin Villalba-Fuentes https://orcid.org/0000-0002-3332-5384

Mayelis Barros-Barrios https://orcid.org/0000-0002-2634-5408

Jeison Muñoz-Montero https://orcid.org/0009-0003-2563-9388

### Data availability

All of the data that support the findings of this study are available in the main text or Supplementary Information.

## Supplementary materials

Supplementary material 1 Data set with ocurrences of Passalidae from the Caribean region of Colombia Data type Ocurrences https://binary.pensoft.net/file/904595This dataset is made available under the Open Database License (http://opendatacommons.org/licenses/odbl/1.0/). The Open Database License (ODbL) is a license agreement intended to allow users to freely share, modify, and use this Dataset while maintaining this same freedom for others, provided that the original source and author(s) are credited.Larry Jiménez-Ferbans, Ana Maestre-Guerra, Evelin Villalba-Fuentes, Mayelis M. Barros-Barrios, Jeison Muñoz-Montero

Supplementary material 2 Label information for the photographed specimens Data type image (JPG file) https://binary.pensoft.net/file/904596This dataset is made available under the Open Database License (http://opendatacommons.org/licenses/odbl/1.0/). The Open Database License (ODbL) is a license agreement intended to allow users to freely share, modify, and use this Dataset while maintaining this same freedom for others, provided that the original source and author(s) are credited.Larry Jiménez-Ferbans, Ana Maestre-Guerra, Evelin Villalba-Fuentes, Mayelis M. Barros-Barrios, Jeison Muñoz-Montero

Supplementary material 3 Spanish version of the key to the Passalidae from the Caribbean Coast of Colombia Data type (Word document) https://binary.pensoft.net/file/904597This dataset is made available under the Open Database License (http://opendatacommons.org/licenses/odbl/1.0/). The Open Database License (ODbL) is a license agreement intended to allow users to freely share, modify, and use this Dataset while maintaining this same freedom for others, provided that the original source and author(s) are credited.Larry Jiménez-Ferbans, Ana Maestre-Guerra, Evelin Villalba-Fuentes, Mayelis M. Barros-Barrios, Jeison Muñoz-Montero

## References

[B1] Amat-García G Fonseca C (1998) Escarabajos pasálidos (Coleoptera: Passalidae) de Colombia. III: Una nueva especie de la Sierra Nevada de Santa Marta. Caldasia 20: 203–206.

[B2] Amat-García G Reyes-Castillo P (2007) Los Passalidae (Coleoptera: Scarabaeoidea) del departamento del Amazonas, Colombia. Caldasia 29: 329–354.

[B3] Amat-García G Blanco-Vargas E Reyes-Castillo P (2004) Lista de especies de los escarabajos pasálidos (Coleoptera: Passalidae) de Colombia. Biota Colombiana 5: 173–182.

[B4] Bevilaqua M (2020) Guide to image editing and production of figures for scientific publications with an emphasis on taxonomy. Zoosystematics and Evolution 96(1): 139–158. 10.3897/zse.96.49225

[B5] Beza-Beza C Jiménez-Ferbans L McKenna D (2020) Phylogeny and Systematics of Wood-Degrading Neotropical Bess Beetles (Coleoptera: Passalidae: Passalinae). Arthropod Systematics & Phylogeny 78: 287–308. 10.26049/ASP78-2-2020-05

[B6] Beza-Beza C Jiménez-Ferbans L McKenna D (2021) Historical biogeography of New World passalid beetles (Coleoptera, Passalidae) reveals Mesoamerican tropical forests as a centre of origin and taxonomic diversification. Journal of Biogeography 48(8): 2037–2052. 10.1111/jbi.14134

[B7] Boucher S (2006) Évolution et phylogénie des coléoptères Passalidae (Scarabaeoidea) Les taxons du groupe famille la tribu néotropicale des Proculini et son complexe *Veturius*. Annales de la Société Entomologique de France (nouv. Ser.) 41: 239–604. 10.1080/00379271.2005.10697444

[B8] Carvajal-Cogollo J Rangel-Ch JO (2012) Amenazas a la biota y los ecosistemas de la región Caribe de Colombia. In: Rangel-Ch JO (Ed.) Colombia Diversidad Biótica XII: La región Caribe de Colombia.Instituto de Ciencias Naturales, Universidad Nacional de Colombia, Bogotá, 851–878.

[B9] Castillo ML Reyes-Castillo P (2003) Los Passalidae: coleópteros tropicales degradadores de troncos de árboles muertos. In: Álvarez-Sánchez J, Naranjo-García E (Eds) Ecología del suelo en la selva tropical húmeda de México, UNAM-Instituto de Ecología, México, 237–262.

[B10] Gillogly A (2005) Review of the genus *Popilius* and preliminary phylogeny of Passalidae (Coleoptera). PhD Thesis, Texas A&M University, College Station, Texas.

[B11] Hernández-Camacho J Hurtado A Ortiz R Walschburger T (1992) Unidades biogeográficas de Colombia. In: Halffter G (Ed.) La diversidad biológica de Iberoamérica I.Instituto de Ecología. A. C. Xalapa, México, 153–173.

[B12] Jiménez-Ferbans L Amat-García G (2009) Sinopsis de los Passalidae (Coleoptera: Scarabaeoidea) del Caribe colombiano. Caldasia 31: 115–173.

[B13] Jiménez-Ferbans L Amat-García G (2010) Clave para los géneros y especies de Passalidae (Coleoptera: Scarabaeoidea) del Caribe colombiano. Intropica 5: 57–62.

[B14] Jiménez-Ferbans L Reyes-Castillo P (2014) Phylogeny, biogeography and description of *Ameripassalus*, a new Mesoamerican genus of Passalidae (Coleoptera). Invertebrate Systematics 28: 124–144. 10.1071/IS13009

[B15] Jiménez-Ferbans L Amat-García G Reyes-Castillo P (2010) Diversity and distribution patterns of Passalidae (Coleoptera: Scarabaeoidea) in the Caribbean Region of Colombia. Tropical Zoology 23: 147–164.

[B16] Jiménez-Ferbans L Amat-García G Reyes-Castillo P (2012) Nueva especie de *Passalus* Fabricius, 1792 (Coleoptera: Scarabaeoidea: Passalidae) de la Sierra Nevada de Santa Marta, Colombia. Acta Zoológica Mexicana (n. s.) 28: 607–612. 10.21829/azm.2012.283862

[B17] Jiménez-Ferbans L Reyes-Castillo P Schuster JC Salazar-Niño K (2013) A checklist and key for the identification of bess beetles (Coleoptera: Passalidae) of Argentina. Zootaxa 3701: 192–206. 10.11646/zootaxa.3701.2.4 26191578

[B18] Jiménez-Ferbans L Reyes-Castillo P Amat-García G (2014) Tres especies colombianas nuevas de Passalidae (Coleoptera: Scarabaeoidea). Revista Mexicana de Biodiversidad 85(1): 31–37. 10.7550/rmb.40501

[B19] Jiménez-Ferbans L Reyes-Castillo P Schuster JC (2015) Passalidae (Coleoptera: Scarabaeoidea) of the Greater and Lesser Antilles. Zootaxa 3956(4): 491–512. 10.11646/zootaxa.3956.4.3 26248935

[B20] Jiménez-Ferbans L González D Reyes-Castillo P (2016) Phylogeny and species delimitation in the group Rhodocanthopus of the genus *Passalus* (Coleoptera: Passalidae) inferred from morphological and molecular data, with description of two new species. Arthropod Systematics & Phylogeny 74: 255–266.

[B21] Jiménez-Ferbans L Reyes-Castillo P Schuster JC Beza-Beza C (2017) The passalid beetles (Coleoptera: Passalidae) from Costa Rica, with the description of two new species of *Passalus*. Revista Mexicana de Biodiversidad 88(3): 608–615. 10.1016/j.rmb.2017.07.016

[B22] Jiménez-Ferbans L Amat-García GD Reyes-Castillo P (2018a) Estudio de los escarabajos pasálidos (Coleoptera: Passalidae) de Colombia. In: Deloya C Gasca-Alvarez H (Eds) Escarabajos del Neotropico (Insecta: Coleoptera), México D.F., 81–245.

[B23] Jiménez-Ferbans L Reyes-Castillo P Schuster JC (2018b) Passalidae (Coleoptera: Scarabaeoidea) of the Biogeographical Province of Chocó and the West Andean Region of Colombia, with the Description of Two New Species. Neotropical Entomology 47(5): 642–667. 10.1007/s13744-017-0584-1 29532447

[B24] Jiménez-Ferbans L Reyes-Castillo P Bevilaqua M (2022) The Brachypterous species of Passalus (Pertinax) (Coleoptera: Passalidae), with the description of a new species from Sierra Nevada de Santa Marta, Colombia. Neotropical Entomology 51(5): 722–741. 10.1007/s13744-022-00988-1 36129624 PMC9546795

[B25] Jiménez-Ferbans L Beza-Beza C Marshall CJ Reyes-Castillo P (2023) Phylogeny of the Neotropical wood degrading beetles (Scarabaeoidea: Passalidae) of the tribe Passalini, inferred from molecular and morphological data. Insect Systematics & Evolution 54 193–214. 10.1163/1876312X-bja10038

[B26] Kuwert A (1891) Systematische Uebersicht der Passaliden-Arten und Gattungen. Deutsche Entomologische Zeitschrift 1: 161–192. 10.1002/mmnd.48018910135

[B27] Marshall CJ (2000) The taxonomy, phylogeny and biogeography of the Neotropical genus, *Verres* Kaup (Coleoptera: Passalidae, Proculini). PhD Thesis, Cornell University. Ithaca, NY.

[B28] Poveda IC Rojas-P Rudas-Ll A Rangel-Ch JO (2004) El Chocó Biogeográfico: Ambiente físico. In: Rangel-Ch JO (Ed.) Colombia - Diversidad Biótica IV.El Chocó biogeográfico/Costa Pacífica. Instituto de Ciencias Naturales. Bogotá, 1–21.

[B29] Reyes-Castillo P (1970) ColeopteraPassalidae: morfología y división en grandes grupos: géneros americanos. Folia Entomologica Mexicana 20: 1–240.

[B30] Reyes-Castillo P (1973) Passalidae de la Guyana Francesa (Coleoptera, Lamellicornia). Bulletin du Muséum National d’Histoire Naturelle 197: 1541–1587.

[B31] Reyes-Castillo P Amat-García GD (2003) Passalidae (Coleoptera) de Colombia. In: Onore G Reyes-Castillo P Zunino M (Eds) Escarabajos de Latinoamérica: estado del conocimiento.Monografías tercer milenio vol. III, Sociedad Entomológica Aragones (SEA), Zaragoza, 35–50.

[B32] Schuster JC (1978) Biogeographical and Ecological Limits of New World Passalidae (Coleoptera). Coleopterists Bulletin 32: 21–28.

[B33] Taboada-Verona C Murillo-Ramos L (2020) The bess beetles (Coleoptera, Passalidae) of three subregions of the department of Sucre, Caribbean region of Colombia. Check List 16(6): 1581–1590. 10.15560/16.6.1581

