# Supplementary figures and images for: ﻿Passalidae (Coleoptera, Scarabaeoidea) from the Caribbean coast of Colombia: synopsis, key, and new species description
Source: Zookeys. 2023 Sep 12;1179:243–97. doi: 10.3897/zookeys.1179.104037 (PMC10509754; doi:10.3897/zookeys.1179.104037)

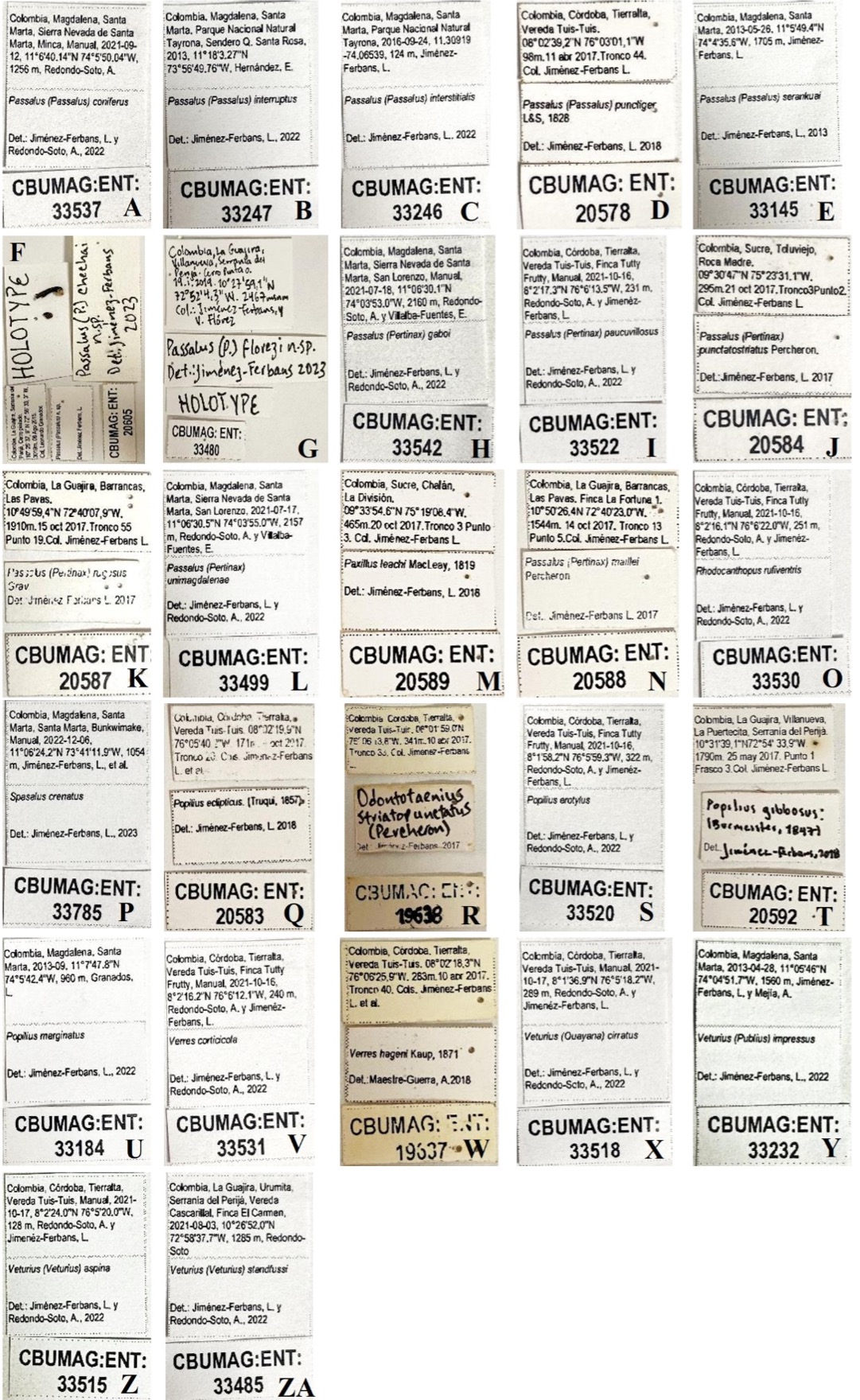

Supplement: Supplementary material 2 — Label information for the photographed specimens [file zookeys-1179-243_article-104037__-s002.jpg]
