# Supplementary material for: ﻿Passalidae (Coleoptera, Scarabaeoidea) from the Caribbean coast of Colombia: synopsis, key, and new species description
Source: Zookeys. 2023 Sep 12;1179:243–97. doi: 10.3897/zookeys.1179.104037 (PMC10509754; doi:10.3897/zookeys.1179.104037)
Supplement: Supplementary material 3 — Spanish version of the key to the Passalidae from the Caribbean Coast of Colombia [file zookeys-1179-243_article-104037__-s003.doc]

**Supplementary file 1**

**Clave para los Passalidae del Caribe colombiano**

1. Clípeo oculto debajo de la frente, con ángulos anteriores por debajo de los tubérculos mediofrontales (Passalini) (Figs 2-19).....................………2

- Clípeo (frontoclípeo) expuesto dorsalmente, con ángulos anteriores por delante del borde anterior de la frente (Proculini) (Figs 20-28)................………………….18

2. Maza antenal con cinco lamelas (Figs 16, 19). Maxila con lacinia unidentada en el tercio apical…..3

- Maza antenal con tres lamelas de longitud similar (Figs 2, 3) o una cuarta lamela (basal, Fig. 4) tomentosa y distintivamente más corta que las otras (*Passalus interstitialis*). Maxila con lacinia bidentada en el tercio apical……5

3. Prosternelo pentagonal, con ápice posterior amplio (Fig. 16C). Fémur I sin surco marginal en el borde anterior de su cara ventral (Fig. 16C). Cuerpo aplanado. (17.5–18.8 mm)......*Paxillus leachi*

- Prosternelo romboidal (Fig. 19C). Fémur I con surco marginal en el borde anterior de su cara ventral. Cuerpo robusto. (*Spasalus*)………….3

4. Parte media basal del mentón casi completamente con puntos y sedas. (17.6–18.2 mm)…..*Spasalus* *crenatus*

- Parte media basal del mentón con puntos y sedas solo en si parte posterior. (15.75–16.30 mm)…..*Spasalus* *paulinae*

5. Tubérculos internos secundarios sobre quillas frontales presentes (Fig. 17A). Ojos reducidos. Meso y metatibia con fuertes espinas en el borde externo (Fig. 17B). Talla pequeña (14.0–19.8 mm). (*Rhodocanthopus*)..............6

- Tubérculos internos secundarios sobre quillas frontales ausentes (Fig. 3A). Ojos no reducidos. Meso y metatibia sin fuertes espinas en el borde externo (Fig. 3B). Talla variable, usualmente mayor a 30 mm. (*Passalus*)........7

6. Cuerpo robusto. Terguitos abdominales de color negro en adultos maduros. (16.3–19.8 mm)...*Rhodocanthopus maillei*

- Cuerpo aplanado. Terguitos abdominales de color rojizo (incluso en adultos maduros). (14.0–16.5 mm)…*Rhodocanthupus rufiventris*

7. Borde anterior de la frente con dos tubérculos mediofrontales secundarios (Fig. 4A). Si el borde es recto, entonces el tubérculo central es de ápice muy libre (Fig. 7A). (Subgenus *Passalus*).......8

- Borde anterior de la frente recto o casi recto, sin tubérculos mediofrontales secundarios. Tubérculo central siempre con ápice no libre (Figs 12A, 14A). (Subgenus *Pertinax*).......14

8. Tubérculo central con ápice muy libre, alcanzando o sobrepasando el borde anterior de la frente (Figs 6A, 9A)…9

- Tubérculo central con ápice no libre o ligeramente libre, no alcanzando el borde frontal anterior (Figs 2D, 3D 4A)……….…11

9. Cuerpo esbelto y aplanado. Macróptero (Fig. 6D). Humeri pubescentes (Fig. 6B). (28.7–34.2 mm)….*Passalus serankuai*

-Cuerpo robusto, subcylindrico (Figs 7D, 9D). Hemi o braquiptero. Humeri pubescencetes basalmente…..10

10. Borde anterior de la frente sin muesca central y sin tubérculos mediofrontales secundarios (Figs 7A, 8C). Tubérculo central sin surco en parte posterior (Fig. 7A). Prosternelo sin surco longitudinal (Fig. 8B). Humeri pubescentes (Fig. 7D). (30.1 mm)…*Passalus chechai* **n.sp.**

- Borde anterior de la frente con muesca central y rudimentarios tubérculos mediofrontales secundarios (Fig. 10C). Tubérculo central con surco en parte posterior (Figs 9A, 10C). Prosternelo con surco longitudinal (Fig. 10B). Humeri con escasas sedas basalmente (Fig. 9D). (36.7–37.8 mm)…*Passalus florezi* **n.sp.**

11. Cuerpo aplanado. Tubérculo central con ápice no libre (Fig. 4D). Maza antenal con cuatro lamelas, la cuarta reducida y tomentosa (Fig. 4A). (23.6–29.9 mm)....... *Passalus* *interstitialis*

- Cuerpo robusto. Tubérculo central con ápice ligeramente libre (Fig. 5D). Maza antenal con solo tres lamelas (Fig. 5A).……12

12. Tubérculo central con ápice claramente libre, pero corto (Fig. 2A, D). Mesosternón con abundante pubescencia, extendiéndose más allá de las cicatrices mesosternales (Fig. 2C). (39.6–46.5 mm)………...*Passalus coniferus*

- Tubérculo central con ápice apenas libre (Fig. 5D). Mesosternón glabro o con escasas sedas en las cicatrices mesosternales (Figs 4C, 5C)…………..13

13. Último esternito abdominal con surco incompleto (Fig. 3C). Talla grande (41.2–52.7 mm)........*Passalus interruptus*

- Último esternito abdominal con surco completo (Fig. 5C). Talla media a grande. (28.4–41.7 mm)........*Passalus punctiger*

14. Humeri y epipleura con pubescencia densa (Fig. 14C, D). (20.1–23.2 mm).....*Passalus rugosus*

- Humeri y epipleura glabros o con unas pocas sedas en la base (Figs 12C, D, 15C, D)………15

15. Fosas frontales con algunas sedas largas esparcidas (Fig. 12A). (22.9–25.5 mm)..........*Passalus paucuvillosus*

- Fosas frontales glabras (Fig. 13A)…….16

16. Ojos reducidos (Fig. 11A). Hemibraquíptero. Mesosternón sin cicatrices mesosternales, señaladas únicamente por un área opaca (Fig. 11C). Talla grande. (31.6–34.2 mm).......*Passalus gaboi*

- Ojos no reducidos (Figs 13A, 15A). Macróptero. Mesosternón con fuertes cicatrices mesosternales (Figs 13C, 15C). Talla pequeña a mediana. (19.5–26.0 mm).......17

17. Tubérculos internos grandes, con ápice libre (Fig. 15A). Humeri con largas sedas en la base (Fig. 15C). (19.5–22.4 mm).....*Passalus unimagdalenae*

- Tubérculos internos, con ápice no libre (Fig. 13A). Humeri glabros (Fig. 13C, D). (21.8–26.0 mm).....*Passalus punctatostriatus*

18. Clípeo ensanchado anteriomedialmente (Fig. 21A). Tubérculo central grande, con ápice libre (Fig. 21D). (28.8 mm).......*Odontotaenius striatopunctatus*

- Clípeo no ensanchado anteriomedialmente (Figs 20A, 22A). Tubérculo central pequeño, con ápice ligeramente libre o no libre (Figs 20A, D, 22A, D)…………19

19. Borde anterior del labro profundamente cóncavo, con una excavación por detrás de la concavidad (depresión dorsal *sensu* Marshall 2000) (Figs 25A, 26A) (*Verres*)........20

- Borde anterior del labro recto o ligeramente cóncavo o convexo, sin excavación por detrás del borde (Figs 24A, 27A)……..21

20. Tubérculos internos grandes, con ápice libre, proyectándose hacia adelante, sobrepasando el frontoclípeo (Fig. 25A, B). Mesosternón opaco (Fig. 25D). (32.9 mm).………*Verres corticicola*

- Tubérculos internos pequeños y romos, no sobrepasando el frontoclípeo (Fig. 26A). Mesosternón brillante (Fig. 26C). (34.4–38.5 mm)……*Verres hageni*

21. Sutura frontoclipeal fuerte, bien definida (Figs 20A, 24A). Borde anterior del pronoto recto (Figs 20A, 24A, B)…..22

- Sutura frontoclipeal ausente (Figs 27A, 28A). Borde anterior del pronoto marcadamente sinuoso (Fig. 30A, B) (*Veturius*)…..25

22. Pubescencia metasternal restringida a la cavidad mesocoxal y foseta lateral (Fig. 20C). Último esternito abdominal rugoso y tuberculado medialmente (Fig. 20C). (28.7–32.5 mm)..............*Heliscus eclipticus*

- Pubescencia metasternal ausente (*Popilius erotylus*, Fig. 22C) o restrictingida a la cavidad mesocoxal y a la parte anterior de la foseta lateral (*Popilius marginatus*, Fig. 24C) o extendida más allá de la cavidad mesocoxal y la foseta lateral (*Popilius gibbosus* Fig. 23C). Último esternito abdominal liso y no tuberculado medialmente (Figs 23C, 24C)…………..23

23. Pubescencia metasternal extendida más allá de la cavidad mesocoxal y la foseta lateral (Fig. 23C). (21.4–23.5 mm)……*Popilius gibbosus*

- Pubescencia metasternal ausente o escasa (restringida a cavidad mesocoxaly parte anterior de foseta) (Figs 22C, 24C)………24

24. Ápice del tubérculo central al nivel de los tubérculos parietales (Fig. 24A, B). (20.6–24.4 mm)…….*Popilius marginatus*

- Ápice del tubérculo central retrasado con respecto al nivel de los tubérculos parietales (Fig. 22A). (23.1–25.0 mm)……*Popilius erotylus*

25. Tubérculos lateroposteriores ausentes (Fig. 28A). Parte posterior de la foseta metasternal menos ancha que la mesotibia (Fig. 28C). Braquíptero. (40.6–47.9 mm)…..*Veturius impressus*

- Tubérculos lateroposteriores presentes (Figs 27A, 29A). Parte posterior de la foseta metasternal al menos del mismo ancho que la mesotibia (Figs 27C, 29C). Macróptero…….26

26. Fosas frontales pubescentes, la pubescencia se extiende sobre las quillas supraorbitarias (Fig. 27A). Mesosternón pubescente (Fig. 27C). Élitros opalescentes (Fig. 27B). (28.7–33.6 mm)........*Veturius* *cirratus*

- Fosas frontales glabras (Fig. 29A) o con escasas sedas (Fig. 30A), nunca extendiéndose sobre las quillas supraorbitarias. Mesosternón glabro posteriormente (Figs 29C, 30C). Élitros no opalescentes (Figs 29B, 30B)..…27

27. Fosas frontales glabras (Fig. 29A). Mesotibia y metatibia sin espinas laterales (Fig. 29B, C). (47.5–50.4 mm)…..*Veturius* *aspina*

- Fosas frontales con escasas sedas (Fig. 30A). Mesotibia y metatibia con pequeñas espinas laterales (Fig. 30B, C). (41.4–46.5 mm).....*Veturius* *standfussi*
